# Modeling the predictors of stunting in Ethiopia: analysis of 2016 Ethiopian demographic health survey data (EDHS)

**DOI:** 10.1186/s40795-020-00378-z

**Published:** 2020-09-22

**Authors:** Hayelom Gebrekirstos Mengesha, Hassan Vatanparast, Cindy Feng, Pammla Petrucka

**Affiliations:** 1 School of Public Health, University of Saskatchewan, Saskatoon, SK Canada; 2 Adigrat University, College of Medicine and Health Sciences, Adigrat, Ethiopia; 3 College of Pharmacy and Nutrition, University of Saskatchewan, Saskatoon, SK Canada; 4 College of Nursing, University of Saskatchewan, Saskatoon, SK Canada; 5 Adjunct Nelson Mandela African Institute of Science and Technology, Arusha, Tanzania

**Keywords:** EDHS, Ethiopia, Stunting, Nutrition, Modeling

## Abstract

**Background:**

Despite continued efforts to address malnutrition, there is minimal reduction in the prevalence rates of stunting in developing countries, including Ethiopia. The association between nutritional and socioeconomic factors collected from a national survey in Ethiopia and stunting have not been rigorously analyzed. Therefore, this study aims to model the effect of nutritional and socioeconomic predictors using 2016 Ethiopian Demographic Health Survey (EDHS) data.

**Methods:**

This study is a secondary data analysis of the 2016 EDHS survey, which included 7909 children aged 6 to59 months. Descriptive statistics using frequency and percentage for categorical data and mean and standard deviation for metric data were conducted. Linearity, confounding, and multicollinearity were checked. Bivariable and multivariable logistic regression were carried out. The adjusted odds ratio (AOR) and 95% confidence interval (CI) were calculated. A receiver operative curve was built to estimate the sensitivity and specificity of the model.

**Results:**

The study identified that 39.2% of children included in this analysis were stunted. Furthermore, 76.47, 84.27, and 92.62% of the children did not consume fruits and vegetables, legumes and lentils, or meat and its products, respectively. Children aged 24 months to 59 months were found to be at 9.71 times higher risk of being stunted compared to their younger counterparts aged 6–24 months (AOR: 9.71; CI: 8.07, 11.6 children). Those children weighing below 9.1 kg were at 27.86 odds of being stunted compared to those weighing 23.3 kg and above. Moreover, mothers with a height below 150 cm (AOR: 2.01; CI: 1.76, 2.5), living in a rural area (AOR: 1.3, CI: 1.09, 1.54), and being male (AOR: 1.4; CI: 1.26, 1.56) were factors associated with stunting. The predictive ability of the model was 77%: if a pair of observations with stunted and non-stunted children were taken, the model correctly ranks 77% of such pair of observations.

**Conclusion:**

The model indicates that being born male, being from a mother of short stature, living in rural areas, small child size, mother with mild anemia, father having no formal education or primary education only, having low child weight, and being 24–59 months of age increases the likelihood of stunting. On the other hand, being born of an overweight or obese mother decreases the likelihood of stunting.

## Background

Nutrition in children, compared to other populations, is critical because childhood is an intense period of mental, physical, and cognitive growth and is highly predictive of future health. Childhood nutrition also reflects long- and short-term negative health consequences, such as reduced economic potential, delayed cognitive development, and educational achievement, along with the increased risk of metabolic syndrome and poor birth outcomes [1–6]. However, macronutrient and micronutrient deficiencies are common during childhood [7] with stunting, wasting, and underweight being the most common nutritional problems affecting millions of children worldwide [8]. According to World Health Organization (WHO) definition, children with height-to-age z-score below minus two standard definition (−2SD) from the median WHO reference population are stunted and those below -3SD are considered to be severely stunted [9]. Chronic malnutrition is highly linked to a lack of adequate nutrient intake during the most critical periods of growth and development of children (i.e. first one thousand days) [10].

Stunting is a global nutritional challenge that disproportionately affects developing nations [8]. It affects 151 million children worldwide, with over 94% of these cases occurring in Asia and Sub-Saharan Africa (SSA) [8]. According to the 2016 Ethiopian Demographic Health Survey [EDHS], the prevalence of stunting is 38% [11]. However, not only is the burden of stunting significant, but it has proven to be resistant to resolution showing slow reduction and a nearly constant rate in Ethiopia between 2016 and 2019 [12]. This high prevalence of stunting has far-reaching implications at the individual, community, societal, and national levels.

The factors associated with stunting are diverse and vary across different geographic, ethnic, and social settings and sectors of societies [13–27]. A systematic review and meta-analysis of 21 studies in Africa found that the predictors of stunting were complementary feeding practices, maternal under-nutrition, household food insecurity, economic growth, and maternal education, [13]. In Ethiopia, studies identified different risk factors associated with stunting [14–17].

A study in northern Ethiopia found that being female, belonging to the age group of 25 to 59 months, having a birth weight of < 2.5 kg, lacking antenatal visits, and mistiming of complementary feeding initiation were positively associated with childhood stunting; whereas increasing educational status of the mother showed negative association [14]. However, another study in northeast Ethiopia identified the factors associated with stunting as being male, increasing age, large family size, poor wealth status, illiterate mother, leftover food, living in a rural area, and experiencing less frequent feedings [15]. In addition, a study in northwest Ethiopia revealed that increased age of the child, and family size of six and above were positively associated with stunting, while fathers with secondary school education, farmers as household heads, and self-employed parents as household head were found to be preventive factors [16]. A rigorous analysis of the 2011 EDHS [28] did not include nutritional factors and the predictors that might vary through time [19]. Most studies respecting stunting are cross-sectional with small sample sizes and in specific localities, which is limiting in fully understanding the breadth and complexity of this issue. Therefore, the aim of this study is to determine the predictors associated with stunting in children of 6 months to 59 months using 2016 EDHS data and a best fit model in order to inform national efforts being made to address stunting syndrome in Ethiopia.

## Methods

### Participants, study setting and procedures

The data from 2016 EDHS was collected from January 2016 to June 2016 in nine geographic regions and two administrative cities of Ethiopia. The survey collects data on demographic and health indicators of all household members with specific emphasis on maternal and child health issues. The sampling frame is based on the Ethiopian Population and Housing Census conducted by the Central Statistical Agency in 2007. The sampling frame is a complete list of 84,915 Enumeration Areas (EA), with each EA comprised of 181 households. Sampling was stratified and conducted at two levels. Each region was stratified into urban and rural, producing 21 strata. Sample EA were selected independently from each stratum in two stages by using proportional allocation and implicit stratification.

Accordingly, in the first stage, 202 in urban areas and 443 in rural areas(a total of 645 EAs) were selected with probability proportional to EA size (based on the 2007 Population and Housing Census) and with independent selection in each sampling stratum. In the second stage of selection, a fixed number of 28 households with an equal probability of systematic selection from the newly created list of households per cluster were selected. Detailed sampling procedures and household selection are found elsewhere [11].

### Data collection tool

Five questionnaires were utilized in the 2016 EDHS - the Household Questionnaire, the Woman’s Questionnaire, the Man’s Questionnaire, the Biomarker Questionnaire, and the Health Facility Questionnaire. All the five questionnaires are presented as an appendix in EDHS 2016 publication [11]. The questionnaires were developed in conjunction with the Demographic Health Survey (DHS), which are customized to an Ethiopian context. Since the DHS recodes the original data into different databases, in this study, we used the children’s recode with the intention to focus our study on children aged 6 to 59 months.

The children’s recode contained data mainly about the households’ sociodemographic and other attributes of the children, mothers/caretakers/primary guardians, and fathers/husbands, as well as nutritional, environmental, and health service-related characteristics.

### Study variables

In this study, relevant variables were selected based on previous literature reviews, subject matter knowledge, and the objective of the study, which was determining a best fit model of the nutritional and background factors associated with stunting. For model building, to determine factors associated with stunting, the maximum model was specified by considering a thoughtful causal diagram, reducing the number of predictor variables based on descriptive statistics, conducting correlations analysis to remove highly correlated variables, and creation of indices for select variables and conducting bivariable analysis. In addition, variables with more than 15% missing values were only described and not included in model building, the effect of continuous variables was also examined, and continuous predictors were tested for linearity.

Based on the above screening procedure, from the sociodemographic and maternal characteristics education of both husband/partner, respondent/ mother highest level of education, age of mother both in a continuous and grouped form, wealth index, residence, sex of child, time to get drinking water, and maternal body mass index (BMI(kg/m^2^)), weight(kg), and height(cm) and anemia. Lightweight SECA with a digital screen designed and manufactured under the guidance of UNICEF mother-infant scales were used to measure weight. A Shorr measuring board was used to measure height of children. Children younger than 24 months were measured for height while lying down, and older children were measured while standing.

Anemia was measured in terms of hemoglobin level in grams/deciliter. Anemia in children is categorized as follows:

1. Non-anemic: Number of children whose hemoglobin count is 11 g per deciliter (g/dl) or higher.
2. Mild anemia: Number of children whose hemoglobin count is between 10.0 and 10.9 g per deciliter (g/dl).
3. Moderate anemia: Number of children whose hemoglobin count is between 7.0 and 9.9 g per deciliter (g/dl).
4. Severe anemia: Number of children whose hemoglobin count is less than 7.0 g per deciliter (g/dl).

Details of data collection procedure on the measurement of blood hemoglobin and anemia categorization for children and women are presented in EDHS 2016 [11].

Child size is the mother’s subjective estimate of baby’s size at the time of birth in the 5 years before the survey. By Percent distribution of births by the size of baby at birth, child size is classified into: very small, smaller than average, average, larger and don’t know/missing. This estimate was obtained because birth weight is unknown for most (86%) newborns in Ethiopia [11].

Education was categorized into no education, primary, secondary, and tertiary. Age of the respondents was initially categorized primarily into seven 5-year groups, but, during analysis, it was re-categorized into four groups (15–19, 20–29, 30–39, 40–49).

The wealth index indicates a composite measure of a household’s cumulative living standard. It is calculated using easy-to-collect data on a household’s ownership of selected assets, such as televisions and bicycles, materials used for housing construction; and types of water access and sanitation facilities. Wealth index was classified into five groups: poorest, poorer, middle, richer, and richest [11]. However, for the sake of analysis, wealth was re-categorized into 3 groups: poor, average, and rich. The details of construction are attached as supplementary material (S1).

Time to get drinking (potable) water was in a continuous form but categorized into less than 30 min or above or equal to 30 min walk. The details of EDHS variables code, including the transformations we made in this study and other details were explained as supplement file.

Regarding the dietary intake of children, the data was based on a 24-h recall (day and night before the interview) by the mother who was asked if she had a child living with her who was born after 2014. If her response was affirmative, the mother was asked if she gave the child certain food group selections. Based on this response, we created five groups according to WHO indicators [29] which included: Fruits and vegetables; Grains, roots and tubers; Legumes and lentils; Dairy products; Meat and its products. Table 1 shows the details of categorization and is attached as a supplement file.

**Table 1:** Sociodemographic and economic characteristics of households (*N* = 7909)

Variable	Frequency	%
**Age of mother at delivery**		
15–19	240	3.03
20–29	3933	49.73
30–39	3065	38.75
40–49	671	8.48
**Time to access clean water (*n*** **= 6503)**		
< 30 min	3748	57.63
≥ 30 min	2755	42.37
Missing	1406	
**Paternal educational attainment**		
No education	3592	48.21
Primary only	2484	33.34
Secondary	754	10.20
Tertiary	564	7.63
**Age child(in months)**		
6 to 24	2919	36.91
> 24 to 59	4990	63.09
**Residence**		
Urban	1452	18.36
Rural	6457	81.64
**Maternal educational attainment**		
No education	5092	64.38
Primary only	2021	25.55
Secondary	513	6.49
Tertiary	283	3.58
**Wealth index**		
Poorest	2821	35.67
Poorer	1396	17.65
Middle	1161	14.68
Richer	987	12.48
Richest	1544	19.52
**Sex of child**		
Male	4454	51.26
Female	3855	48.74
**Birth interval**		
< 24 month	1533	24.14
≥ 24 month	4817	75.86
Missing	1559	

### Data management and analysis

Data were cleaned and analyzed in SAS™ 9.4. Categorical variables were described using frequency and percentages. For continuous variables, mean and standard deviation (SD) were used. Cross-tabulations between some predictor and outcome variables were conducted to check assumptions. Histograms and quartiles were used to present data. For ordinal variables, we used the Spearman correlation. For continuous variables, we used the Pearson correlation to check the correlation between independent predictors, with r ≥ 0.7 used as the cut-off value for correlation. Multi-collinearity was checked using the variance inflation factor (VIF) with VIF < 2.5 used as a cut-off point. Interaction and confounders were tested. The interaction was checked among pairs of variables that were suspected of having interactions based on prior knowledge and literature. A bivariable logistic regression analysis using an Unadjusted Odds Ratio (UOR) was carried out to select candidate variables with *P*-values of < 0.25 for multivariable logistic regression model building. In multivariable logistic regression analysis model building, backward elimination was used. Finally, variables with a *P-*value of 0.05 with 95% confidence interval (CI) and adjusted odds ratio (AOR) were conducted. A receiver operative characteristics curve (ROC) with sensitivity and specificity was also depicted to determine the predictive ability of the model. Model goodness-of-fit was assessed by using the Hosmer and Lemeshow test. Linearity was assessed by comparing the squared variable with un-squared variable significance; if the squared variable was significant, the variable was classified into plausible categories. A model selection for non-nested models was done using Akaike’s Information Criteria (AIC), and a model with smaller values was selected.

## Results

### Participants’ sociodemographic summary

In the original recoded children data, there were10641 children under five and 1209 variables. On rigorous data cleaning and management process, 32 variables and 7909 children with mothers were included in the analysis.

### Sociodemographic characteristics of 6–59 months old children

Regarding the place of residence, more than 80% of the children resided in rural areas. Nearly half of the husbands/partners had no education, and 4217(52%) were categorized in the poor category of wealth status. See Table 1 for details.

### Description of nutritional characteristics of children aged 6–59 months

The prevalence of stunting in this population was 39.1%. A few (10%) children were given dairy products, which included yogurt and cheese, by their mothers/caretakers. The most common foods eaten were grains and tubers, which were consumed by almost half of the children. Less than a quarter of children consumed fruits and vegetables (23.53%), with the lowest food group consumed being meat (7.38%) and its products followed by legumes and lentils (15.73%). There were large missing value sets for this factor because mothers/caretakers were only asked for children born in 2014 or later and limited to one child per household. See details in Table 2.

**Table 2:** Nutritional and clinical characteristics of mothers and children 6–59 months

Variable	Freq.	%
**Stunting**		
Yes	3101	39.21
No	4808	60.79
**Dairy products (*n*** **= 4770)**		
No	3929	82.36
Yes	841	17.64
Missing	3139	
**Grains, roots, and tubers (Gratube) (*n*** **= 4770)**		
No	2333	48.9
Yes	2437	51.1
Missing	3139	
**Fruits and vegetables (Fruveg) (*n*** **= 4770)**		
No	3648	76.47
Yes	1122	23.53
Missing	3139	
**Meat and its products (*n*** **= 4770)**		
No	4418	92.62
Yes	352	7.38
Missing	3139	
**Legumes and lentils (*n*** **= 4770)**		
Yes	750	15.73
No	4020	84.27
Missing	3139	
**Size of child**		
Large	2419	30.85
Average	3353	42.77
Small	2068	26.38
Missing	69	
**Maternal height**		
< 150 cm	776	9.81
> =150 cm	7133	90.19
**Anemia level of the mother (*n*** **= 7609)**		
Severe	126	1.64
Moderate	730	9.48
Mild	1795	23.32
Not anemic	5046	65.56
**BMI**^**a**^ **of mother**		
< 18.5	1921	24.29
18.5–24.9	5174	65.42
≥ 25	722	9.13
Missing	92	1.16
**Anemia level of the child**		
Severe	300	3.94
Moderate	2455	32.22
Mild	1812	23.78
Not anemic	3052	40.06
Missing	290	
**Weight of child**		
< 9.1 kg^b^	1948	2463
≥ 9.1–11.1 kg	1964	2483
≥ 11.2–13.3 kg	2005	2535
> 13.3 kg	1992	2519

^**a**^**Body mass index;** ^**b**^**kilogram**

### Description of continuous variables

The average age of women who participated in the survey was 29.4 (6.5) years, and the average weight of the child during the survey was 11.3(2.8) kg (see Table 3 for details).

**Table 3:** Mean and standard deviation of continuous variables of children 6–59 months old in Ethiopia of 2016 EDHS

Variable	Mean	SD^**b**^
Age of mother (*n* = 7909) in years	29.45	6.5
Time to access clean water (*n* = 6503) in minutes	57.93	75.84
Number of children (*n* = 7909)	1.85	0.81
Weight mother (*n* = 7820) in kg	51.7	9.29
Height mother (*n* = 7821) in m	1.58	0.68
BMI^a^ mother (*n* = 7817) in kg/m^2^	20.66	3.34
Weight child (*n* = 7907) in kg	11.3	2.8
Height of child (*n* = 7909) in m	0.85	0.116

^a^Body mass index;^b^standard deviation

### Correlation and linearity of independent variables

Simple Pearson correlation was conducted for continuous variables. However, Spearman correlation was used for ordered categorical variables. Based on these tests significant correlation was not obtained. The correlation was considered significant if r was above 70%. Based on this level, there was no significant correlation among the independent variables.

The linearity of selected independent variables was checked with weight of the child squared found to be significant in the model. The quadratic form of the variable was significant and considering this, the interaction term was included in the final model; however, it created instability in the model due to high collinearity with the normal variable (unsquared weight of the child). Therefore, the weight of the child was categorized into quartiles.

### Bivariable analysis and multi-collinearity

The bivariable analysis showed that maternal factors including height, anemia level, education, and BMI; child factors including age, anemia level, weight, sex, and size; paternal education; and place of residence (urban/rural) were significantly associated with stunting **(see** Table 4**).**

**Table 4:** Bi-variable analysis and cross-tabulation of the outcome variable versus the covariates in children of age 6–59 months using 2016 EDHS dataset

Variable	Stunting		UOR	95%CI	***P***-value
	No, freq(%)	Yes, freq(%)			
**Age of mother**					
15–19	138(1.74)	102(1.29)	ref	ref	
20–29	2404(30.4)	1529(19.33)	1.16	0.89,1.51	0.22
30–39	1866(23.59)	1199(15.16)	1.15	0.88,1.5	0.33
40–49	400(5.06)	271(3.43)	1.09	0.80,1.47	0.91
Overall *p-*value = 0.643					
**Maternal height**					
≥ 150 cm	4461(56.4)	2672(33.78)	ref	ref	ref
< 150 cm	347(4.39)	429(5.42)	2.06	1.77–2.39	< 0.0001
**Anemia level of the mother**					
Severe	78(1.01)	48(0.62)	1.008	0.7,1.45	0.96
Moderate	428(5.56)	302(3.92)	1.15	0.98,1.35	0.07
Mild	1036(13.46)	759(9.86)	1.2	1.07,1.33	0.0011
Not anemic	3133(40.7)	1913(24.85)	ref	ref	ref
**Anemia level of child**					
Severe	135(1.77)	165(2.17)	2.2	1.73,2.79	< 0.0001
Moderate	1425(18.7)	1030(13.52)	1.3	1.16,1.45	< 0.0001
Mild	1075(14.11)	737(9.67)	1.23	1.09,1.39	0.0006
Non-anemic	1962(25.75)	1090(14.31)	ref	ref	ref
Overall *P-*value = 0.0069					
**Child size**					
Large	1608(20.51)	811(10.34)	ref	ref	
Average	2059(26.26)	1294(16.51)	1.24	1.11,1.39	< 0.0001
Small	1099(14.02)	969(12.36)	1.74	1.54,1.97	< 0.0001
**Paternal education**					
No education	1993(26.95)	1599(21.63)	2.87	2.33,3.55	< 0.0001
Primary	1537(20.79)	947(12.81)	2.2	1.78,2.74	< 0.0001
Secondary	525(7.10)	229(3.10)	1.56	1.21,2.01	0.0005
Tertiary	441(5.96)	123(1.66)	ref	ref	
**Maternal education**					
No education	2899(36.65)	2193(27.73)	4.22	3.03,5.86	< 0.0001
Primary	1281(16.26)	740(9.36)	3.22	2.3,4.5	< 0.0001
Secondary	388(4.91)	125(1.58)	1.79	1.22,2.63	0.0026
Tertiary	240(3.03)	43(0.54)	ref	ref	
**Age child**					
6 to 24 months	2017(25.5)	902(11.4)	ref	ref	
≥ 24 to 59	2791(35.29)	2199(27.8)	1.76	1.6,1.94	< 0.0001
**Wealth index**					
Poor	2321(29.35)	1896(23.97)	1.98	1.78,2.2	< 0.0001
Average	695(8.79)	466(5.890	1.62	1.4,1.88	< 0.0001
Rich	1792(22.66)	739(9.34)	ref	ref	
**Sex of child**					
Male	2400(30.35)	1654(20.91)	1.14	1.04,1.25	0.003
Female	2408(30.45)	1,447,918.3)	ref	ref	
**Residence**					
Urban	1076(13.6)	376(4.75)	ref	ref	
Rural	3732(47.19)	2725(34.45)	2.09	1.84,2.37	< 0.0001
**BMI of mother**					
< 18.5	1094(14)	827(10.58)	1.13	1.02,1.26	0.018
≥ 18.5–24.9	3107(39.75)	2067(26.44)	ref	ref	
≥ 25	556(7.11)	166(2.12)	0.44	0.37,0.53	< 0.0001
**Weight child**					
< 9.1	1117(14.12)	830(10.51)	3.81	3.28,4.42	
≥ 9.1–11.2	956(12.09)	1008(12.74)	5.4	4.66,6.27	
≥ 11.2–13.3	1068(13.5)	937(11.85)	4.5	3.88,5.21	
> 13.3	1667(21.08)	325(4.11)	ref	ref	
**Continuous variables**	**B-coefficient CI**		***P-*value**		
Age of mother	1.003	0.997,1.01	0.325		
Weight of child	0.983	0.981, 0.985	0.0001		
Height of mother	0.995	0.994,0.995	< 0.0001		

*UOR* unadjusted odds ratio, *Freq* frequency, *CI* confidence interval

Based on restrictive multi-collinearity test values of ≥2.5, there was no multi-collinearity between the independent variables found in this study.

### Predictors of stunting

After a rigorous screening, steps were taken to select candidate variables, such as mothers’ education because of a small number of observation in the third category and moderate correlation with husband education, all children food intake history (fruits and vegetables, grains, tubers and roots, lentils and legumes, meat groups and dairy products), birth intervals, and number of children were excluded because of missing values. Based on this screening, after wealth and anemia of the child were excluded during model building, while maternal height, child size, paternal education, age and sex of child, residence, BMI of the mother, anemia level of the mother, and weight of child were all independent predictors of stunting in our study.

Mothers who were below 150 cm tall were at a 2.01 times increased risk of having stunted children compared to mothers who were 150 cm or above (AOR: 2.01; CI: 1.76, 2.5). Age of the child and weight of the child during the interview were strong factors associated with stunting. Children aged 24 to 59 months were at a 9.71 times higher risk of being stunted compared to younger children aged 6 to 24 months old (AOR: 9.71; CI: 8.07,11.6). Similarly, children weighing less than 9.1 kg were at 27.86 odds of being stunted compared to those weighing 23.3 kg and above. From the sociodemographic determinants, living in a rural area increases the odds of stunting by 30% than living in the urban areas (AOR: 1.3; CI: 1.09, 1.54). From the unavoidable factors, sex of the child, being male, increase the odds of stunting by 40% than their female counterparts (AOR: 1.4; CI: 1.26, 1.56)[see Table 5 for details].

**Table 5:** Multivariable analysis of variables included in the final model of children age between 6 months and 59 months included in 2016 EDHS

Variable	AOR	95%CI
**Maternal height**		
≥ 150 cm	Ref	Ref
< 150 cm	2.01	1.76,2.5
**Child size**		
Large	Ref	ref
Average	1.12	0.98,1.27
Small	1.38	1.19,1.59
**Paternal education**		
No education	1.79	1.39,2.3
Primary	1.62	1.25,2.1
secondary	1.32	0.99,1.77
tertiary	Ref	ref
**Age child**		
6 to 24 months	Ref	ref
> 24 to 59 months	9.71	8.07,11.6
**Sex of child**		
Male	1.4	1.26,1.56
Female	ref	ref
**Residence**		
Urban	ref	ref
Rural	1.3	1.09,1.54
**BMI of mother**		
< 18.5	1.02	0.9, 1.16
≥ 18.5–24.9	ref	ref
≥ 25–29.9	0.69	0.54,0.89
≥ 30	0.59	0.37, 0.94
**Anemia level of mother**		
Severe	0.73	0.48,1.11
Moderate	0.92	0.76,1.11
Mild	1.14	1.1,1.3
Not anemic	ref	ref
**Weight of child**		
< 9.1	27.8	21.8,35.38
9.1 to 11.2	15.64	12.91,18.95
11.2 to 13.3	5.19	4.41,6.10
> 13.3	ref	ref

***AOR*** **Adjusted odds ratio,** ***CI*** **confidence interval,** ***BMI*** **Body mass index**

#### Result of interaction test in the final model

There was no significant interaction found among the independent factors affecting stunting. Interaction was tested based on previous literature and knowledge of the subject matter. Interaction was tested between age of the child and weight of the child, education and anemia level, height of the mother and BMI.

#### Goodness of fit of the model

The model developed in this study fits the data well (*P* = 0.85). We choose the Hosmer and Lemeshow goodness fit test because our study contained too many categories, and the number of unique profiles were 1823.

#### Predictive ability of the model

##### Comparing different non-nested models

**Fig. 1:**
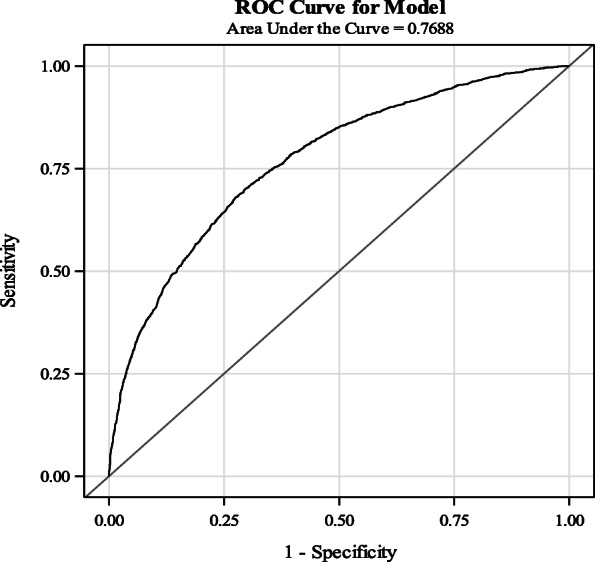
Receiver operating characteristics curve for the model developed on stunting predictors. The area under the curve (AUC) in our model was found to be 0.7688 which is equals to 77%. This means a randomly selected individual from the stunting group has a true test value larger than that for a randomly chosen individual from the non-stunted group 77% of the time. Such that if a pair of observations with stunted and non-stunted children were taken the model ranks 77% of such pairs of observations correctly

Non-nested models were compared using AIC value for select continuous variables like the weight of the child versus the categorical weight of child; the height of mother in meters versus categorized height of the mother, and age of the child in months versus categorical age in months. We chose a model with the smaller AIC value which, in this case, was the categorized form of the variables (Fig. 1).

## Discussion

The aim of this study was to model the predictors of stunting using a dataset that is representative of the nation, and the findings could possibly be put in to practice. To the best of our knowledge, this is the first study that analyzed the 2016 EDHS and considered nutritional factors and dietary patterns, developed a model, and rigorously tested for its fitness. The model will open the door for future studies to be conducted and improve the Area under the curve (AUC), sensitivity and specificity of the model by including other important factors that are not measured in this study. Therefore, the prevalence of stunting in included children of 6 to 59 months found to be 39.1%. More importantly, we found that children aged above 24 months, low weight, small size, being male at birth, short maternal stature, overweight and obese maternal status, rural residence, no education and primary education of husband, and mild anemia of the mother were predictors of stunting. Among this child age, low weight, and maternal stature were the strongest predictors with AOR above 2.

Child age above 24–59 months was strongly associated with stunting with the AOR above 2. A number of studies have found similar associations [14, 17, 19]. This evidence corroborates that stunting is malnutrition, which starts during pregnancy and continues until the second year of life with the most frequent appearance after the second year of life [6]. However, this finding does not mean intervening after the second year of life is not effective as there are numerous immediate avoidable factors that, if intervened, may reduce the effects of stunting after the second year of life. Studies have shown that factors associated with stunting in late childhood and adolescence are different, and there are other windows of opportunities to stop chronic malnutrition if the first 1000 years window is missed [30]. Regardless, our study finding reaffirms that, to reduce stunting, intervention strategies ideally will focus on the child before 24 months. Therefore, policies and interventions should focus on the first one thousand days to prevent the stunting incidence. Equally important, the period between 24 and 59 months is crucial to provide a personalized intervention, such as providing specific nutritional needs for children with stunting, to accelerate catch-up growth.

The current weight of the child was the strongest predictor of stunting, according to the 2016 EDHS data. The continuous form of the variable was shown in a non-linear relationship (quadratic) with stunting. Consequently, the variable is categorized into quartiles. There are no previous studies that assessed the effect of child weight on stunting directly, although studies have shown that wasting (weight to height) is associated with stunting [22, 23], low birth weight is associated with stunting [14], and weight to-age are indicators which reflect cumulative effects of wasting and stunting [11]. The association between child weight and stunting could be very strong because there are a few children with a large weight and being stunted at the same time; hence, this could inflate the association. Children’s weights reflect body composition with a recent study finding that stunted mothers have low body composition, including small kidneys and other organs, are thin, and give birth to small infants [31]. The implication of this finding is that weight monitoring of children is critical in preventing and intervening in cases of stunting. Prospective future studies need to assess the effectiveness of weight monitoring overtime on stunting incidence.

Maternal stature was one of the independent predictors of stunting. Different study findings were in line with our findings [21, 27]. Maternal stature reflects the intergenerational effects of stunting, maternal malnutrition, and its consequences on childhood outcomes [6]. This knowledge suggests that focusing on mothers of short stature might help in improving birth outcomes and in following-up of children after birth. However, the maternal stature cut-off point we used was arbitrary as there is no universally agreed cut-off point in the literature. Intuitively, the cut-off point should be locally derived as genetics may also have a role in determining one’s height. This observation could be similarly applied to stunting’s definition which might not be a perfect indicator of malnutrition as linear growth failure may be caused by different biological causes apart from inadequate nutrition [32].

In addition, maternal BMI, rural residence, paternal education, being male, and child’s size were important independent predictors. These findings are similar to those of recent literature [14, 15, 19, 20, 24]. Although there is some contrasting evidence regarding the association of sex with stunting [14], most studies’ findings indicated male children are more likely to be affected by stunting [15, 16, 19, 26], which could be due to biological or metabolic differences across sexes [33]. A meta-analysis of 16 demography health surveys conducted in SSA concluded that male children under 5 years of age are more likely to become stunted than females, which might suggest that boys are more vulnerable to health inequalities than their female counterparts in the same age groups [34]. Further studies are required to uncover the reason behind this finding. Policies and interventions that on stunting reduction need to place a due emphasis on gender differentials.

Education is also a well-established factor for the likelihood stunting. Previous studies have found a similar association [19, 26]. Stunting is a complex problem correlated with the socioeconomic level of a society and a nation at large. Achieving the sustainable development goals is addressing the problem of stunting indirectly [35]. However, since stunting affects generations, hundreds of millions of individuals are already stunted, so intervention strategies should focus not only first 1000 years but also on these different population groups to stop the stunting syndrome as there might be other windows of opportunities for intervention [30].

The prevalence of stunting has decreased by about 1% every year from 58% in 2000 to 38% in 2016 [11, 36] and unexpectedly has shown almost no reduction between 2016 and 2019 at 37% [11, 12]. It has increased in some regions of Ethiopia, such as in Tigray region; it has increased by almost 10% from 38% in 2016 to 48.7% 2019 [11, 12]. This probably indicates the failure to design workable policies and locally based interventions. Particularly, data on dietary intake were not collected and analyzed in previous demographic surveys. Therefore, this study gives an important insight to design programs and policies based on the local evidence to prevent and address stunting.

The main limitation of this study is missing data on some variables, especially with respect to our intent to include all nutritional factors, including dietary habits which was limited due to missing data (almost 40%) in the EDHS data set. Multiple imputation was beyond the scope this analysis. Moreover, AUC of the model we developed is not high because of missing or unmeasured variables which indicate the need for future supportive studies to improve its predictive ability. However, it evident from the descriptive statistics that, on average, very few (i.e., one in five of children) received adequate and diverse nutrition which included grains, animal and plant-based proteins, fruits and vegetables.

### Conclusion

Our model indicates that being born male, being from a mother of short stature, living in a rural area, small child size, mother with mild anemia, father with no education or primary education only, low weight of the child, and over 24 months of age increases the likelihood of stunting. On the other hand, being born to an overweight or obese mother decreases the likelihood of stunting.

The model fits the data very well with an AUC of 77%. Improving the nutritional and socioeconomic status of women pre-pregnancy and during pregnancy might reduce the burden of stunting.

Future studies are needed which focus on determining the association between body composition of the mother, medical conditions, and childhood stunting, finding another window of opportunity to minimize/reverse the consequences in already stunted children, and impact of low child weight should receive due emphasis on future endeavors of reducing chronic malnutrition. Moreover, we recommend the DHS to include more robust, and a contextually customized objective dietary/ nutritional patterns questionnaires in upcoming surveys.

## Supplementary information

**Additional file 1: Table 1.** EDHS variable codes, and explanation of variables included to this study.

**Additional file 2.**

## Data Availability

The data we analyzed in this study are publicly available at https://www.dhsprogram.com/data/dataset/Ethiopia_Standard-DHS_2016.cfm?flag=0.

## Abbreviations

AUC: Area under the curve
AIC: Akaike information criteria
BMI: Body mass index
EDHS: Ethiopian demographic health survey
ROC: Receiver operating curve
SD: Standard deviation
SSA: Sub-Saharan Africa
VIF: Variance inflation factor
