# Supplementary material for: Modeling the predictors of stunting in Ethiopia: analysis of 2016 Ethiopian demographic health survey data (EDHS)
Source: BMC Nutr. 2020 Sep 22;6:52. doi: 10.1186/s40795-020-00378-z (PMC7507682; doi:10.1186/s40795-020-00378-z)
Supplement: Supplementary file 1 — Additional file 1: Table 1. EDHS variable codes, and explanation of variables included to this study. [file 40795_2020_378_MOESM1_ESM.docx]

**Table 1: EDHS variable codes, and explanation of variables included to this study**

| **Variable type** | **Variables code EDHS(if applicable)** | **Explanation** | **Type of variable** | **Newly Categorized** | **New variable name in this study** |
| --- | --- | --- | --- | --- | --- |
| **Outcome** | HW70 | Height-to-age of the children between age of 6 month and 59 months. | Nominal | Stunting, Yes, yes(1)/no(0) | Stunting |
| **Main factors and confounders** | V106 | Highest education of women | Nominal | No education(0), primary(1), secondary(2), tertiary(3 | Maternal education |
|  | V012 | Age of the respondent continuous | Scale(interval) | No | Age of mother |
|  | V013 | Age of respondent in 5-year space | Nominal | Yes, 15-19(1),20-29(2),30-39(3),40-49(4) | Age of mothercat |
|  | V025 | Type of place of residence | Nominal | No, urban(1), rural(2) | residence |
|  | V115 | Time to get drinking water | Scale | Yes, <30 min(1),≥30min(2) | Time to get water |
|  | V137 | Number of children(de jure) | Scale | Yes, one(1), two to four(2), ≥five(3) | Children alive |
|  | V190 | Wealth index | Nominal | Poor(1), average(2), rich(3) | Wealth index |
|  | V437 | Weight of mother | Scale | NO | NA |
|  | V438 | Height of mother | Scale | Yes, <150cm(1),≥150cm(2) | Maternal height |
|  | V439 | Height/age percentile of mother | Scale | No | NA |
|  | V445 | BMI of mother | Scale | Yes,<18.5(1), 18.5-24.9=(2), 25-29.9(3),≥30(4) | BMI |
|  | V457 | Anemia level of mother | Nominal | No, severe(1), moderate(2), mild(3), no anemia(4) | Anemia of mother |
|  | V701 | Husband/partner educational level | Nominal | No | Husedu |
|  | B4 | Sex of child | Nominal | male(1), female(2) | Sex |
|  | B11 | Preceding birth interval | Scale | Yes, <24=1,24=2 | Birth interval |
|  | M18 | Size of the child | Nominal | Yes, Large(1), medium(2), small==(3) | Size child |
|  | HW1 | Child age in month | Scale | Yes,6-24 month(1), ≥24-59 month(2) | Age child |
|  | HW2 | Child weight in kilogram | Scale | NO | NA |
|  | HW3 | Child height in centimeter | Scale | NO | NA |
|  | HW57 | Anemia level of child | Nominal | No, severe(1), moderate(2),mild(3), No anemia(4) | Anemia of child |
|  | HW70 | Height/age SD new (WHO) | Scale | Yes,≤2sd(1_,≥2sd(0) | Stunting |
|  | HW2 | Child weight | Scale | <9.1kg(1), 9.1 to 11.1kg(2),11.1 to 13.3kg(3),13.3kg(4) |  |
|  | V414I | Gave child pumpkin, carrot, squash | Nominal | Yes(1), No(0) | NA |
|  | V414J | Gave child any dark green leafy vegetables | Nominal | Yes(1), No(0) | NA |
|  | V414K | Gave child mangoes, papaya and other vitamin A fruits | Nominal | Yes(1), No(0) | NA |
|  | V414L | Gave child any other fruit | Nominal | Yes(1), No(0) | NA |
|  | V414V | Gave child yogurt | Nominal | Yes(1), No(0) | NA |
|  | V414F | Gave child potato, cassava, other tubers | Nominal | Yes(1), No(0) | NA |
|  | V414O | Gave child made from beans, lentils and nuts | Nominal | Yes(1), No(0) | No |
|  | V414E | Gave child noodles, bread and other grains | Nominal | Yes(1), No(0) | NA |
|  | V414P | Gave child cheese,yogurt, | Nominal | Yes(1), No(0) | NA |
|  | V414M | Gave child liver, heart,other organs | Nominal | Yes(1), No(0) | NA |
|  | V414N | Gave child fish, shellfish | Nominal | Yes(1), No(0) | NA |
|  | N414H | Gave beef, pork lamb meat etc.. | Nominal | Yes(1), No(0) | NA |
| Newly categorized variables | Dairy product | If the child took any dairy product by 24-recall | Nominal | Yes(1), No(0) | NA |
|  | gratube | If the mother gave tubers, roots and grains | Nominal | Yes(1), No(0) | NA |
|  | fruveg | If the mother gave any fruits and vegetables | Nominal | Yes(1), No(0) | NA |
|  | meat | If the mother gave any meat and, and its products | Nominal | Yes(1), No(0) | NA |
