# Supplementary material for: Modeling the predictors of stunting in Ethiopia: analysis of 2016 Ethiopian demographic health survey data (EDHS)
Source: BMC Nutr. 2020 Sep 22;6:52. doi: 10.1186/s40795-020-00378-z (PMC7507682; doi:10.1186/s40795-020-00378-z)
Supplement: Supplementary file 2 — Additional file 2. [file 40795_2020_378_MOESM2_ESM.pdf]

Common

Descriptive Statistics

	Mean	Std. Deviation[a]	Analysis N[a]	Missing N
QH101_11 Source of drinking water: Piped into dwelling	.02	.139	16650	0
QH101_12 Source of drinking water: Piped to yard/plot	.19	.395	16650	0
QH101_13 Source of drinking water: Piped to neighbor	.05	.225	16650	0
QH101_14 Source of drinking water: Public tap/standpipe	.14	.352	16650	0
QH101_21 Source of drinking water: Tube well or borehole	.13	.340	16650	0
QH101_31 Source of drinking water: Protected well	.06	.243	16650	0
QH101_32 Source of drinking water: Unprotected well	.06	.235	16650	0
QH101_41 Source of drinking water: Protected spring	.08	.269	16650	0
QH101_42 Source of drinking water: Unprotected spring	.12	.329	16650	0
QH101_51 Source of drinking water: Rainwater	.01	.072	16650	0
QH101_61 Source of drinking water: Tanker truck	.01	.078	16650	0
QH101_71 Source of drinking water: Cart with small tank	.00	.066	16650	0
QH101_81 Source of drinking water: Surface water (river/dam/lake/pond/stream/canal/irrigation channel)	.11	.309	16650	0
QH101_91 Source of drinking water: Bottled water	.01	.088	16650	0
QH101_96 Source of drinking water: Other	.00	.034	16650	0
QH109_11 Type of toilet facility: Flush to piped sewer system	.01	.086	16650	0
QH109_12 Type of toilet facility: Flush to septic tank	.01	.109	16650	0
QH109_13 Type of toilet facility: Flush to pit latrine	.01	.114	16650	0
QH109_14 Type of toilet facility: Flush to somewhere else	.00	.033	16650	0
QH109_15 Type of toilet facility: Flush, don't know where	.00	.031	16650	0
QH109_21 Type of toilet facility: Ventilated improved pit latrine	.00	.051	16650	0
QH109_22 Type of toilet facility: Pit latrine with slab	.06	.230	16650	0
QH109_23 Type of toilet facility: Pit latrine without slab/open pit	.26	.441	16650	0
QH109_31 Type of toilet facility: Composting toilet	.01	.071	16650	0
QH109_41 Type of toilet facility: Bucket toilet	.00	.019	16650	0
QH109_51 Type of toilet facility: Hanging toilet/hanging latrine	.00	.019	16650	0
QH109_61 Type of toilet facility: No facility/bush/field	.35	.478	16650	0
QH109_96 Type of toilet facility: Other	.00	.036	16650	0
QH109_11_sh Type of toilet facility: Flush to piped sewer system - shared	.00	.043	16650	0
QH109_12_sh Type of toilet facility: Flush to septic tank - shared	.01	.078	16650	0
QH109_13_sh Type of toilet facility: Flush to pit latrine - shared	.01	.121	16650	0
QH109_14_sh Type of toilet facility: Flush to somewhere else - shared	.00	.039	16650	0
QH109_15_sh Type of toilet facility: Flush, don't know where - shared	.00	.038	16650	0
QH109_21_sh Type of toilet facility: Ventilated improved pit latrine - shared	.00	.070	16650	0
QH109_22_sh Type of toilet facility: Pit latrine with slab - shared	.13	.334	16650	0
QH109_23_sh Type of toilet facility: Pit latrine without slab/open pit - shared	.12	.323	16650	0
QH109_31_sh Type of toilet facility: Composting toilet - shared	.00	.041	16650	0
QH109_51_sh Type of toilet facility: Hanging toilet/hanging latrine - shared	.00	.046	16650	0
QH109_96_sh Type of toilet facility: Other - shared	.00	.060	16650	0
QH113_1 Type of cooking fuel: Electricity	.08	.278	16650	0
QH113_2 Type of cooking fuel: LPG	.00	.057	16650	0
QH113_3 Type of cooking fuel: Natural gas	.00	.044	16650	0

Component Score Coefficient Matrix

	Component 1
QH101_11 Source of drinking water: Piped into dwelling	.024
QH101_12 Source of drinking water: Piped to yard/plot	.072
QH101_13 Source of drinking water: Piped to neighbor	.016
QH101_14 Source of drinking water: Public tap/standpipe	-.012
QH101_21 Source of drinking water: Tube well or borehole	-.021
QH101_31 Source of drinking water: Protected well	-.012
QH101_32 Source of drinking water: Unprotected well	-.017
QH101_41 Source of drinking water: Protected spring	-.013
QH101_42 Source of drinking water: Unprotected spring	-.024
QH101_51 Source of drinking water: Rainwater	-.005
QH101_61 Source of drinking water: Tanker truck	.002
QH101_71 Source of drinking water: Cart with small tank	.000
QH101_81 Source of drinking water: Surface water (river/dam/lake/pond/stream/canal/irrigation channel)	-.024
QH101_91 Source of drinking water: Bottled water	.017
QH101_96 Source of drinking water: Other	-.001
QH109_11 Type of toilet facility: Flush to piped sewer system	.022
QH109_12 Type of toilet facility: Flush to septic tank	.025
QH109_13 Type of toilet facility: Flush to pit latrine	.008
QH109_14 Type of toilet facility: Flush to somewhere else	.006
QH109_15 Type of toilet facility: Flush, don't know where	.001
QH109_21 Type of toilet facility: Ventilated improved pit latrine	.010
QH109_22 Type of toilet facility: Pit latrine with slab	.024
QH109_23 Type of toilet facility: Pit latrine without slab/open pit	-.022
QH109_31 Type of toilet facility: Composting toilet	-.004
QH109_41 Type of toilet facility: Bucket toilet	.002
QH109_51 Type of toilet facility: Hanging toilet/hanging latrine	.001
QH109_61 Type of toilet facility: No facility/bush/field	-.050
QH109_96 Type of toilet facility: Other	.000
QH109_11_sh Type of toilet facility: Flush to piped sewer system - shared	.008
QH109_12_sh Type of toilet facility: Flush to septic tank - shared	.014
QH109_13_sh Type of toilet facility: Flush to pit latrine - shared	.013
QH109_14_sh Type of toilet facility: Flush to somewhere else - shared	.006
QH109_15_sh Type of toilet facility: Flush, don't know where - shared	.004
QH109_21_sh Type of toilet facility: Ventilated improved pit latrine - shared	.009
QH109_22_sh Type of toilet facility: Pit latrine with slab - shared	.046
QH109_23_sh Type of toilet facility: Pit latrine without slab/open pit - shared	.008
QH109_31_sh Type of toilet facility: Composting toilet - shared	.002
QH109_51_sh Type of toilet facility: Hanging toilet/hanging latrine - shared	.007
QH109_96_sh Type of toilet facility: Other - shared	.000
QH113_1 Type of cooking fuel: Electricity	.060
QH113_2 Type of cooking fuel: LPG	.006
QH113_3 Type of cooking fuel: Natural gas	.005

Sum over each variable

If has	If does not have
0.17234842	-0.003474213
0.147697623	-0.03545315
0.069114818	-0.003903058
-0.028255547	0.004777374
-0.053893813	0.008287047
-0.045581435	0.00305551
-0.066446844	0.004128555
-0.043375529	0.003710307
-0.064057296	0.009014366
-0.072325014	0.0003799
0.019941231	-0.000121703
-0.003525706	1.53125E-05
-0.070263727	0.008437319
0.187652249	-0.001488132
-0.019177334	2.1909E-05
0.256259953	-0.001938426
0.230498666	-0.002816599
0.072239674	-0.000967299
0.177430218	-0.000192024
0.03477835	-3.34528E-05
0.187809089	-0.000497627
0.100350551	-0.005936769
-0.037168515	0.013292654
-0.05445802	0.00027944
0.081180935	-2.92649E-05
0.055710439	-2.00831E-05
-0.06837247	0.037171905
0.005289615	-6.99853E-06
0.195719346	-0.000365082
0.173100897	-0.0010775
0.102287235	-0.001552925
0.141870526	-0.000221886
0.096329668	-0.000139054
0.125845301	-0.000622846
0.119888317	-0.017530138
0.020965975	-0.002803839
0.040993283	-6.90538E-05
0.145838681	-0.00031601
0.002486286	-8.99199E-06
0.196748586	-0.018076252
0.110353856	-0.00036574
0.118836689	-0.000236

QH113_4 Type of cooking fuel: Biogas	.00	.054	16650	0
QH113_5 Type of cooking fuel: Kerosene	.01	.115	16650	0
QH113_6 Type of cooking fuel: Charcoal	.14	.347	16650	0
QH113_7 Type of cooking fuel: Wood	.69	.463	16650	0
QH113_8 Type of cooking fuel: Straw/shrubs/grass	.00	.052	16650	0
QH113_9 Type of cooking fuel: Agricultural crop	.01	.093	16650	0
QH113_10 Type of cooking fuel: Animal dung	.03	.183	16650	0
QH113_95 Type of cooking fuel: No food cooked in household	.02	.134	16650	0
QH113_96 Type of cooking fuel: Other	.00	.015	16650	0
QH121A Electricity	.36	.480	16650	0
QH121B Radio	.30	.458	16650	0
QH121C Television	.23	.421	16650	0
QH121D Telephone (non-mobile)	.05	.224	16650	0
QH121E Computer	.04	.201	16650	0
QH121F Refrigerator	.11	.310	16650	0
QH121G Table	.35	.477	16650	0
QH121H Chair	.44	.496	16650	0
QH121I Bed with cotton/spring mattress	.39	.488	16650	0
QH121J Electric mitad	.10	.303	16650	0
QH121K Kerosene lamp/ preassure lamp	.09	.283	16650	0
QH122A Watch	.28	.447	16650	0
QH122B Mobile telephone	.61	.487	16650	0
QH122C Bicycle	.03	.163	16650	0
QH122D Motorcycle or scooter	.01	.101	16650	0
QH122E Animal-drawn cart	.01	.119	16650	0
QH122F Car or Truck	.02	.122	16650	0
QH122G Boat with a motor	.00	.042	16650	0
QH122H Bagag	.01	.089	16650	0
QH123 Bank account	.32	.466	16650	0
QH142_11 Main floor material: Earth/sand	.53	.499	16650	0
QH142_12 Main floor material: Dung	.18	.381	16650	0
QH142_21 Main floor material: Wood planks	.00	.041	16650	0
QH142_22 Main floor material: Palm/bamboo	.01	.082	16650	0
QH142_31 Main floor material: Parquet or polished wood	.00	.058	16650	0
QH142_32 Main floor material: Vinyl or asphalt strips	.10	.307	16650	0
QH142_33 Main floor material: Ceramic tiles	.01	.115	16650	0
QH142_34 Main floor material: Cement	.08	.264	16650	0
QH142_35 Main floor material: Carpet	.09	.281	16650	0
QH142_96 Main floor material: Other	.00	.026	16650	0
QH143_11 Main roof material: No roof	.00	.041	16650	0
QH143_12 Main roof material: Thatch/mud	.06	.241	16650	0
QH143_13 Main roof material: Sod	.24	.427	16650	0
QH143_21 Main roof material: Rustic mat	.07	.257	16650	0
QH143_22 Main roof material: Palm/bamboo	.01	.089	16650	0
QH143_23 Main roof material: Wood planks	.02	.131	16650	0
QH143_24 Main roof material: Cardboard	.00	.040	16650	0
QH143_31 Main roof material: Metal/ corrugated iron	.57	.495	16650	0
QH143_32 Main roof material: Wood	.01	.082	16650	0
QH143_33 Main roof material: Calamine/cement fiber	.00	.026	16650	0
QH143_34 Main roof material: Ceramic tiles	.00	.031	16650	0
QH143_35 Main roof material: Cement	.02	.125	16650	0
QH143_36 Main roof material: Roofing shingles	.00	.013	16650	0
QH143_96 Main roof material: Other	.00	.061	16650	0
QH144_11 Main wall material: No walls	.01	.091	16650	0
QH144_12 Main wall material: Cane/palm/trunks	.08	.274	16650	0
QH144_13 Main wall material: Dirt	.02	.129	16650	0
QH144_21 Main wall material: Bamboo with mud	.63	.484	16650	0

QH113_4 Type of cooking fuel: Biogas	.007	0.134321302	-0.000388352
QH113_5 Type of cooking fuel: Kerosene	.015	0.130435602	-0.001786789
QH113_6 Type of cooking fuel: Charcoal	.045	0.110592576	-0.017976506
QH113_7 Type of cooking fuel: Wood	-.074	-0.049623962	0.110375837
QH113_8 Type of cooking fuel: Straw/shrubs/grass	-.002	-0.044228241	0.000122531
QH113_9 Type of cooking fuel: Agricultural crop	-.004	-0.042394242	0.000375034
QH113_10 Type of cooking fuel: Animal dung	-.007	-0.038373954	0.00137263
QH113_95 Type of cooking fuel: No food cooked in household	.009	0.069485352	-0.001287947
QH113_96 Type of cooking fuel: Other	.001	0.081262831	-1.95273E-05
QH121A Electricity	.081	0.108308038	-0.061289332
QH121B Radio	.042	0.064780358	-0.02756493
QH121C Television	.083	0.152026349	-0.045448976
QH121D Telephone (non-mobile)	.046	0.196198942	-0.010935189
QH121E Computer	.046	0.220312561	-0.009697731
QH121F Refrigerator	.068	0.195664373	-0.023584016
QH121G Table	.050	0.067425182	-0.036483489
QH121H Chair	.042	0.048032859	-0.037410208
QH121I Bed with cotton/spring mattress	.065	0.081836073	-0.05234124
QH121J Electric mitad	.068	0.20056002	-0.022791216
QH121K Kerosene lamp/ preassure lamp	.001	0.003910663	-0.000375312
QH122A Watch	.031	0.049939026	-0.01897236
QH122B Mobile telephone	.051	0.040820106	-0.06458541
QH122C Bicycle	.021	0.122819738	-0.003427245
QH122D Motorcycle or scooter	.009	0.092073724	-0.000955435
QH122E Animal-drawn cart	.000	0.001575364	-2.30401E-05
QH122F Car or Truck	.029	0.237142353	-0.003614975
QH122G Boat with a motor	.004	0.105238152	-0.000183617
QH122H Bagag	.013	0.142350386	-0.001154938
QH123 Bank account	.064	0.093937743	-0.044011051
QH142_11 Main floor material: Earth/sand	-.058	-0.054483903	0.06172888
QH142_12 Main floor material: Dung	-.022	-0.047903643	0.010242865
QH142_21 Main floor material: Wood planks	.004	0.101141461	-0.000170374
QH142_22 Main floor material: Palm/bamboo	-.001	-0.009644601	6.64904E-05
QH142_31 Main floor material: Parquet or polished wood	.011	0.191656943	-0.000646787
QH142_32 Main floor material: Vinyl or asphalt strips	.044	0.127730408	-0.014982737
QH142_33 Main floor material: Ceramic tiles	.026	0.225153858	-0.003070404
QH142_34 Main floor material: Cement	.035	0.124266525	-0.010104019
QH142_35 Main floor material: Carpet	.039	0.125296263	-0.011862368
QH142_96 Main floor material: Other	.000	0.016020909	-1.05914E-05
QH143_11 Main roof material: No roof	-.002	-0.051254231	8.63385E-05
QH143_12 Main roof material: Thatch/mud	-.016	-0.063384793	0.004175338
QH143_13 Main roof material: Sod	-.043	-0.076649665	0.024173287
QH143_21 Main roof material: Rustic mat	-.016	-0.058313628	0.004464201
QH143_22 Main roof material: Palm/bamboo	-.006	-0.061071677	0.000495496
QH143_23 Main roof material: Wood planks	-.007	-0.052559581	0.00093822
QH143_24 Main roof material: Cardboard	-.001	-0.029221678	4.74635E-05
QH143_31 Main roof material: Metal/ corrugated iron	.057	0.049623655	-0.065820505
QH143_32 Main roof material: Wood	.001	0.010987827	-7.50816E-05
QH143_33 Main roof material: Calamine/cement fiber	.006	0.220541234	-0.000145799
QH143_34 Main roof material: Ceramic tiles	.007	0.21217185	-0.000204085
QH143_35 Main roof material: Cement	-.004	-0.028512086	0.000461135
QH143_36 Main roof material: Roofing shingles	.000	-0.023276353	4.19469E-06
QH143_96 Main roof material: Other	-.004	-0.071544405	0.000271737
QH144_11 Main wall material: No walls	-.007	-0.077124716	0.000653995
QH144_12 Main wall material: Cane/palm/trunks	-.024	-0.078952709	0.007016986
QH144_13 Main wall material: Dirt	-.010	-0.0798897	0.00136647
QH144_21 Main wall material: Bamboo with mud	-.017	-0.012948953	0.021596803

QH144_22 Main wall material: Stone with mud	.11	.315	16650	0
QH144_23 Main wall material: Uncovered adobe	.00	.051	16650	0
QH144_24 Main wall material: Plywood	.00	.038	16650	0
QH144_25 Main wall material: Cardboard	.00	.035	16650	0
QH144_26 Main wall material: Reused wood	.01	.079	16650	0
QH144_31 Main wall material: Cement	.07	.255	16650	0
QH144_32 Main wall material: Stone with lime/cement	.04	.195	16650	0
QH144_33 Main wall material: Bricks	.00	.047	16650	0
QH144_34 Main wall material: Cement blocks	.01	.101	16650	0
QH144_35 Main wall material: Covered adobe	.00	.056	16650	0
QH144_36 Main wall material: Wood planks/shingles	.01	.074	16650	0
QH144_96 Main wall material: Other	.01	.118	16650	0
DOMESTIC Domestic staff	.00	.064	16650	0
HOUSE Owns a house	.56	.497	16650	0
LAND Owns land	.63	.482	16650	0
memsleep Number of members per sleeping room	3.56	2.151	16650	42

a. For each variable, missing values are replaced with the variable mean.

QH144_22 Main wall material: Stone with mud	-.006
QH144_23 Main wall material: Uncovered adobe	.008
QH144_24 Main wall material: Plywood	-.001
QH144_25 Main wall material: Cardboard	.000
QH144_26 Main wall material: Reused wood	-.006
QH144_31 Main wall material: Cement	.044
QH144_32 Main wall material: Stone with lime/cement	.028
QH144_33 Main wall material: Bricks	.011
QH144_34 Main wall material: Cement blocks	.018
QH144_35 Main wall material: Covered adobe	.003
QH144_36 Main wall material: Wood planks/shingles	-.005
QH144_96 Main wall material: Other	-.003
DOMESTIC Domestic staff	.012
HOUSE Owns a house	-.030
LAND Owns land	-.061
memsleep Number of members per sleeping room	-.037

Extraction Method: Principal Component Analysis.
Component Scores.

-0.01635435	0.002056734
0.15189696	-0.000393302
-0.026513253	3.82725E-05
-0.000785153	9.44261E-07
-0.076196346	0.000478933
0.161332537	-0.012104107
0.138686831	-0.005733441
0.226792801	-0.000505106
0.174805557	-0.001824648
0.055514626	-0.000173922
-0.073641818	0.000404699
-0.025723418	0.000369851
0.189824361	-0.000789933
-0.026581342	0.033715571
-0.046335304	0.079785803
((memsleep-3.56021)/2.15095)*(-0.03692)	

Urban

Descriptive Statistics

	Mean	Std. Deviation	Analysis N[a]	Missing N
QH101_11 Source of drinking water: Piped into dwelling	.06	.235	5232	0
QH101_12 Source of drinking water: Piped to yard/plot	.58	.493	5232	0
QH101_13 Source of drinking water: Piped to neighbor	.15	.355	5232	0
QH101_14 Source of drinking water: Public tap/standpipe	.10	.304	5232	0
QH101_21 Source of drinking water: Tube well or borehole	.02	.127	5232	0
QH101_31 Source of drinking water: Protected well	.01	.121	5232	0
QH101_32 Source of drinking water: Unprotected well	.00	.062	5232	0
QH101_41 Source of drinking water: Protected spring	.02	.140	5232	0
QH101_42 Source of drinking water: Unprotected spring	.01	.086	5232	0
QH101_51 Source of drinking water: Rainwater	.00	.014	5232	0
QH101_61 Source of drinking water: Tanker truck	.01	.078	5232	0
QH101_71 Source of drinking water: Cart with small tank	.01	.090	5232	0
QH101_81 Source of drinking water: Surface water (river/dam/lake/pond/stream/canal/irrigation channel)	.01	.087	5232	0
QH101_91 Source of drinking water: Bottled water	.02	.149	5232	0
QH101_96 Source of drinking water: Other	.00	.031	5232	0
QH109_11 Type of toilet facility: Flush to piped sewer system	.02	.153	5232	0
QH109_12 Type of toilet facility: Flush to septic tank	.04	.185	5232	0
QH109_13 Type of toilet facility: Flush to pit latrine	.02	.148	5232	0
QH109_14 Type of toilet facility: Flush to somewhere else	.00	.059	5232	0
QH109_15 Type of toilet facility: Flush, don't know where	.00	.039	5232	0
QH109_21 Type of toilet facility: Ventilated improved pit latrine	.01	.077	5232	0
QH109_22 Type of toilet facility: Pit latrine with slab	.12	.325	5232	0
QH109_23 Type of toilet facility: Pit latrine without slab/open pit	.09	.292	5232	0
QH109_31 Type of toilet facility: Composting toilet	.00	.024	5232	0
QH109_41 Type of toilet facility: Bucket toilet	.00	.031	5232	0
QH109_51 Type of toilet facility: Hanging toilet/hanging latrine	.00	.024	5232	0
QH109_61 Type of toilet facility: No facility/bush/field	.07	.255	5232	0
QH109_96 Type of toilet facility: Other	.00	.041	5232	0
QH109_11_sh Type of toilet facility: Flush to piped sewer system - shared	.01	.076	5232	0
QH109_12_sh Type of toilet facility: Flush to septic tank - shared	.02	.137	5232	0
QH109_13_sh Type of toilet facility: Flush to pit latrine - shared	.04	.194	5232	0
QH109_14_sh Type of toilet facility: Flush to somewhere else - shared	.00	.069	5232	0

Component Score Coefficient Matrix

	Component
	1
QH101_11 Source of drinking water: Piped into dwelling	.025
QH101_12 Source of drinking water: Piped to yard/plot	.069
QH101_13 Source of drinking water: Piped to neighbor	-.033
QH101_14 Source of drinking water: Public tap/standpipe	-.051
QH101_21 Source of drinking water: Tube well or borehole	-.027
QH101_31 Source of drinking water: Protected well	-.021
QH101_32 Source of drinking water: Unprotected well	-.014
QH101_41 Source of drinking water: Protected spring	-.025
QH101_42 Source of drinking water: Unprotected spring	-.022
QH101_51 Source of drinking water: Rainwater	-.003
QH101_61 Source of drinking water: Tanker truck	-.003
QH101_71 Source of drinking water: Cart with small tank	-.017
QH101_81 Source of drinking water: Surface water(river/dam/lake/pond/stream/canal/irrigation channel)	-.019
QH101_91 Source of drinking water: Bottled water	.018
QH101_96 Source of drinking water: Other	.000
QH109_11 Type of toilet facility: Flush to piped sewer system	.036
QH109_12 Type of toilet facility: Flush to septic tank	.042
QH109_13 Type of toilet facility: Flush to pit latrine	.009
QH109_14 Type of toilet facility: Flush to somewhere else	.005
QH109_15 Type of toilet facility: Flush, don't know where	.001
QH109_21 Type of toilet facility: Ventilated improved pit latrine	.011
QH109_22 Type of toilet facility: Pit latrine with slab	.018
QH109_23 Type of toilet facility: Pit latrine without slab/open pit	-.037
QH109_31 Type of toilet facility: Composting toilet	-.005
QH109_41 Type of toilet facility: Bucket toilet	-.003
QH109_51 Type of toilet facility: Hanging toilet/hanging latrine	.003
QH109_61 Type of toilet facility: No facility/bush/field	-.061
QH109_96 Type of toilet facility: Other	-.002
QH109_11_sh Type of toilet facility: Flush to piped sewer system - shared	.010
QH109_12_sh Type of toilet facility: Flush to septic tank - shared	.014
QH109_13_sh Type of toilet facility: Flush to pit latrine - shared	.003
QH109_14_sh Type of toilet facility: Flush to somewhere else - shared	.004

Sum over each variable

If has	If does not have
0.101425566	-0.006300492
0.05859855	-0.081844699
-0.078471837	0.013583018
-0.149747453	0.017163215
-0.211773658	0.003539163
-0.170967483	0.002587401
-0.22637925	0.000868685
-0.178234595	0.003614742
-0.251148989	0.001886156
-0.190721662	3.64599E-05
-0.043492038	0.000267643
-0.184575463	0.001529533
-0.217600486	0.001676429
0.118744347	-0.002763657
-0.011873649	1.1358E-05
0.228170089	-0.005584739
0.219415164	-0.008042759
0.062043739	-0.001431592
0.092728086	-0.00032012
0.035948785	-5.50517E-05
0.141709321	-0.000844643
0.048875015	-0.006642588
-0.114513882	0.01185959
-0.200480061	0.00011502
-0.099081671	9.47787E-05
0.133525107	-7.66065E-05
-0.220284849	0.016617583
-0.051237972	8.82906E-05
0.13792412	-0.00079541
0.099044527	-0.00192994
0.017112054	-0.000694284
0.059077319	-0.000283644

QH109_15_sh Type of toilet facility: Flush, don't know where - shared	.00	.066	5232	0	QH109_15_sh Type of toilet facility: Flush, don't know where - shared	-.003	-0.038890004	0.000171716
QH109_21_sh Type of toilet facility: Ventilated improved pit latrine - shared	.01	.114	5232	0	QH109_21_sh Type of toilet facility: Ventilated improved pit latrine - shared	.006	0.053240807	-0.000711527
QH109_22_sh Type of toilet facility: Pit latrine with slab - shared	.35	.477	5232	0	QH109_22_sh Type of toilet facility: Pit latrine with slab - shared	.020	0.026808017	-0.014529898
QH109_23_sh Type of toilet facility: Pit latrine without slab/open pit - shared	.17	.373	5232	0	QH109_23_sh Type of toilet facility: Pit latrine without slab/open pit - shared	-.023	-0.05036163	0.010100061
QH109_31_sh Type of toilet facility: Composting toilet - shared	.00	.050	5232	0	QH109_31_sh Type of toilet facility: Composting toilet - shared	.001	0.0196527	-4.89529E-05
QH109_51_sh Type of toilet facility: Hanging toilet/hanging latrine - shared	.01	.083	5232	0	QH109_51_sh Type of toilet facility: Hanging toilet/hanging latrine - shared	.003	0.04041707	-0.000280026
QH109_96_sh Type of toilet facility: Other - shared	.01	.076	5232	0	QH109_96_sh Type of toilet facility: Other - shared	-.010	-0.131163101	0.000756419
QH113_1 Type of cooking fuel: Electricity	.26	.438	5232	0	QH113_1 Type of cooking fuel: Electricity	.083	0.139869212	-0.048835202
QH113_2 Type of cooking fuel: LPG	.01	.077	5232	0	QH113_2 Type of cooking fuel: LPG	.011	0.146542394	-0.00087345
QH113_3 Type of cooking fuel: Natural gas	.01	.077	5232	0	QH113_3 Type of cooking fuel: Natural gas	.001	0.011320551	-6.74749E-05
QH113_4 Type of cooking fuel: Biogas	.01	.093	5232	0	QH113_4 Type of cooking fuel: Biogas	.003	0.02995136	-0.00026567
QH113_5 Type of cooking fuel: Kerosene	.04	.199	5232	0	QH113_5 Type of cooking fuel: Kerosene	.004	0.019284503	-0.000834444
QH113_6 Type of cooking fuel: Charcoal	.38	.485	5232	0	QH113_6 Type of cooking fuel: Charcoal	.002	0.002101748	-0.001285912
QH113_7 Type of cooking fuel: Wood	.25	.434	5232	0	QH113_7 Type of cooking fuel: Wood	-.085	-0.14612235	0.049055627
QH113_8 Type of cooking fuel: Straw/shrubs/grass	.00	.014	5232	0	QH113_8 Type of cooking fuel: Straw/shrubs/grass	-.001	-0.037206869	7.11276E-06
QH113_9 Type of cooking fuel: Agricultural crop	.00	.039	5232	0	QH113_9 Type of cooking fuel: Agricultural crop	-.005	-0.119577834	0.000183121
QH113_10 Type of cooking fuel: Animal dung	.00	.059	5232	0	QH113_10 Type of cooking fuel: Animal dung	-.008	-0.134130192	0.00046305
QH113_95 Type of cooking fuel: No food cooked in household	.04	.202	5232	0	QH113_95 Type of cooking fuel: No food cooked in household	-.008	-0.038346759	0.001699198
QH113_96 Type of cooking fuel: Other	.00	.024	5232	0	QH113_96 Type of cooking fuel: Other	.000	0.019603306	-1.12469E-05
QH121A Electricity	.92	.266	5232	0	QH121A Electricity	.074	0.021255961	-0.256078669
QH121B Radio	.48	.499	5232	0	QH121B Radio	.058	0.06061201	-0.05512596
QH121C Television	.67	.471	5232	0	QH121C Television	.097	0.068587431	-0.137885203
QH121D Telephone (non-mobile)	.16	.363	5232	0	QH121D Telephone (non-mobile)	.060	0.13867617	-0.025736638
QH121E Computer	.13	.335	5232	0	QH121E Computer	.066	0.171309411	-0.025418165
QH121F Refrigerator	.33	.469	5232	0	QH121F Refrigerator	.091	0.13094167	-0.063519533
QH121G Table	.58	.493	5232	0	QH121G Table	.060	0.050678046	-0.070283421
QH121H Chair	.65	.478	5232	0	QH121H Chair	.053	0.038965774	-0.071114642
QH121I Bed with cotton/spring mattress	.75	.435	5232	0	QH121I Bed with cotton/spring mattress	.079	0.046004284	-0.135378092
QH121J Electric mitad	.31	.463	5232	0	QH121J Electric mitad	.094	0.140255413	-0.063469274
QH121K Kerosene lamp/ preassure lamp	.07	.261	5232	0	QH121K Kerosene lamp/ preassure lamp	.007	0.024355136	-0.001934542
QH122A Watch	.39	.487	5232	0	QH122A Watch	.057	0.072015316	-0.045619443
QH122B Mobile telephone	.90	.301	5232	0	QH122B Mobile telephone	.053	0.017776527	-0.159042467
QH122C Bicycle	.06	.230	5232	0	QH122C Bicycle	.023	0.094731072	-0.005640125
QH122D Motorcycle or scooter	.02	.129	5232	0	QH122D Motorcycle or scooter	.009	0.069555905	-0.00120367
QH122E Animal-drawn cart	.01	.116	5232	0	QH122E Animal-drawn cart	-.013	-0.113374314	0.001559693
QH122F Car or Truck	.04	.203	5232	0	QH122F Car or Truck	.046	0.217753694	-0.009785217
QH122G Boat with a motor	.00	.050	5232	0	QH122G Boat with a motor	.005	0.09936624	-0.000247511
QH122H Bagag	.02	.136	5232	0	QH122H Bagag	.011	0.077203472	-0.001473693
QH123 Bank account	.66	.474	5232	0	QH123 Bank account	.073	0.052558064	-0.101667281
QH142_11 Main floor material: Earth/sand	.20	.400	5232	0	QH142_11 Main floor material: Earth/sand	-.084	-0.167937291	0.041964263
QH142_12 Main floor material: Dung	.03	.176	5232	0	QH142_12 Main floor material: Dung	-.033	-0.178590011	0.005924787
QH142_21 Main floor material: Wood planks	.00	.059	5232	0	QH142_21 Main floor material: Wood planks	.007	0.120239063	-0.000415095
QH142_22 Main floor material: Palm/bamboo	.00	.046	5232	0	QH142_22 Main floor material: Palm/bamboo	-.010	-0.210213233	0.000442893
QH142_31 Main floor material: Parquet or polished wood	.01	.099	5232	0	QH142_31 Main floor material: Parquet or polished wood	.014	0.143590857	-0.001441453
QH142_32 Main floor material: Vinyl or asphalt strips	.30	.457	5232	0	QH142_32 Main floor material: Vinyl or asphalt strips	.022	0.033814392	-0.014221692
QH142_33 Main floor material: Ceramic tiles	.04	.198	5232	0	QH142_33 Main floor material: Ceramic tiles	.040	0.192743919	-0.008219848
QH142_34 Main floor material: Cement	.19	.392	5232	0	QH142_34 Main floor material: Cement	.021	0.044256706	-0.010328651
QH142_35 Main floor material: Carpet	.23	.418	5232	0	QH142_35 Main floor material: Carpet	.028	0.051695569	-0.015054485

QH142_96 Main floor material: Other	.00	.028	5232	0	QH142_96 Main floor material: Other	-.001	-0.023438918	1.79334E-05
QH143_11 Main roof material: No roof	.00	.028	5232	0	QH143_11 Main roof material: No roof	-.006	-0.20025023	0.000153214
QH143_12 Main roof material: Thatch/mud	.02	.134	5232	0	QH143_12 Main roof material: Thatch/mud	-.025	-0.182269684	0.00340691
QH143_13 Main roof material: Sod	.03	.163	5232	0	QH143_13 Main roof material: Sod	-.050	-0.298330488	0.008322776
QH143_21 Main roof material: Rustic mat	.03	.177	5232	0	QH143_21 Main roof material: Rustic mat	-.017	-0.090905375	0.003052926
QH143_22 Main roof material: Palm/bamboo	.00	.037	5232	0	QH143_22 Main roof material: Palm/bamboo	-.008	-0.206430372	0.000276557
QH143_23 Main roof material: Wood planks	.01	.083	5232	0	QH143_23 Main roof material: Wood planks	-.005	-0.058403014	0.00040464
QH143_24 Main roof material: Cardboard	.00	.041	5232	0	QH143_24 Main roof material: Cardboard	-.005	-0.110438636	0.000190302
QH143_31 Main roof material: Metal/ corrugated iron	.89	.315	5232	0	QH143_31 Main roof material: Metal/ corrugated iron	.044	0.015667982	-0.12446002
QH143_32 Main roof material: Wood	.01	.091	5232	0	QH143_32 Main roof material: Wood	.001	0.01065279	-9.03475E-05
QH143_33 Main roof material: Calamine/cement fiber	.00	.044	5232	0	QH143_33 Main roof material: Calamine/cement fiber	.008	0.181208772	-0.00034701
QH143_34 Main roof material: Ceramic tiles	.00	.055	5232	0	QH143_34 Main roof material: Ceramic tiles	.008	0.13615876	-0.000417665
QH143_35 Main roof material: Cement	.01	.094	5232	0	QH143_35 Main roof material: Cement	.007	0.07580877	-0.000687177
QH143_36 Main roof material: Roofing shingles	.00	.014	5232	0	QH143_36 Main roof material: Roofing shingles	.000	-0.020293328	3.87944E-06
QH143_96 Main roof material: Other	.00	.024	5232	0	QH143_96 Main roof material: Other	-.001	-0.051326168	2.9447E-05
QH144_11 Main wall material: No walls	.00	.039	5232	0	QH144_11 Main wall material: No walls	-.005	-0.133115947	0.000203853
QH144_12 Main wall material: Cane/palm/trunks	.01	.090	5232	0	QH144_12 Main wall material: Cane/palm/trunks	-.021	-0.227260815	0.001883256
QH144_13 Main wall material: Dirt	.00	.065	5232	0	QH144_13 Main wall material: Dirt	-.018	-0.271015152	0.001144402
QH144_21 Main wall material: Bamboo with mud	.54	.498	5232	0	QH144_21 Main wall material: Bamboo with mud	-.050	-0.046158362	0.054508811
QH144_22 Main wall material: Stone with mud	.08	.266	5232	0	QH144_22 Main wall material: Stone with mud	-.003	-0.009325906	0.000774102
QH144_23 Main wall material: Uncovered adobe	.01	.082	5232	0	QH144_23 Main wall material: Uncovered adobe	.010	0.122859024	-0.000827413
QH144_24 Main wall material: Plywood	.00	.034	5232	0	QH144_24 Main wall material: Plywood	.000	0.00826653	-9.49085E-06
QH144_25 Main wall material: Cardboard	.00	.044	5232	0	QH144_25 Main wall material: Cardboard	-.005	-0.114404746	0.000219082
QH144_26 Main wall material: Reused wood	.00	.014	5232	0	QH144_26 Main wall material: Reused wood	-.003	-0.235272123	4.49765E-05
QH144_31 Main wall material: Cement	.20	.400	5232	0	QH144_31 Main wall material: Cement	.045	0.089075658	-0.022311494
QH144_32 Main wall material: Stone with lime/cement	.10	.301	5232	0	QH144_32 Main wall material: Stone with lime/cement	.024	0.072282966	-0.008113394
QH144_33 Main wall material: Bricks	.01	.082	5232	0	QH144_33 Main wall material: Bricks	.016	0.189076984	-0.001273368
QH144_34 Main wall material: Cement blocks	.03	.173	5232	0	QH144_34 Main wall material: Cement blocks	.018	0.102244999	-0.003246193
QH144_35 Main wall material: Covered adobe	.01	.088	5232	0	QH144_35 Main wall material: Covered adobe	-.006	-0.06541168	0.00051664
QH144_36 Main wall material: Wood planks/shingles	.00	.020	5232	0	QH144_36 Main wall material: Wood planks/shingles	-.005	-0.253125324	9.67974E-05
QH144_96 Main wall material: Other	.01	.105	5232	0	QH144_96 Main wall material: Other	-.006	-0.052969797	0.000593786
DOMESTIC Domestic staff	.01	.108	5232	0	DOMESTIC Domestic staff	.014	0.130870874	-0.001569438
HOUSE Owns a house	.33	.470	5232	0	HOUSE Owns a house	-.008	-0.011790153	0.005754238
LAND Owns land	.19	.394	5232	0	LAND Owns land	-.046	-0.094403724	0.022362301
memsleep Number of members per sleeping room	2.6	1.684	5232	36	memsleep Number of members per sleeping room	-.037	((memsleep-2.63587)/1.68370)*(-0.03681)	
QH118A_1 Cows/bulls: 1-4	.0470	.21170	5232	0	QH118A_1 Cows/bulls: 1-4	-.045	-0.201615422	0.009947331
QH118A_2 Cows/bulls: 5-9	.0084	.09133	5232	0	QH118A_2 Cows/bulls: 5-9	-.021	-0.222695195	0.001888703
QH118A_3 Cows/bulls: 10+	.0050	.07033	5232	0	QH118A_3 Cows/bulls: 10+	-.015	-0.208251712	0.001040058
QH118B_1 Other cattle: 1-4	.0294	.16904	5232	0	QH118B_1 Other cattle: 1-4	-.042	-0.241913174	0.007336477
QH118B_2 Other cattle: 5-9	.0027	.05166	5232	0	QH118B_2 Other cattle: 5-9	-.011	-0.21550838	0.000578213
QH118B_3 Other cattle: 10+	.0006	.02394	5232	0	QH118B_3 Other cattle: 10+	-.004	-0.165907071	9.51848E-05
QH118C_1 Horses/donkeys/mules: 1-4	.0245	.15450	5232	0	QH118C_1 Horses/donkeys/mules: 1-4	-.038	-0.237374757	0.005952972
QH118D_1 Camels: 1-4	.0019	.04368	5232	0	QH118D_1 Camels: 1-4	-.014	-0.331287602	0.000634408
QH118D_2 Camels: 5-9	.0002	.01383	5232	0	QH118D_2 Camels: 5-9	-.008	-0.584091998	0.00011166
QH118D_3 Camels: 10+	.0008	.02764	5232	0	QH118D_3 Camels: 10+	-.004	-0.15686657	0.00012002
QH118E_1 Goats: 1-4	.0369	.18851	5232	0	QH118E_1 Goats: 1-4	-.030	-0.153126057	0.005864919
QH118E_2 Goats: 5-9	.0119	.10822	5232	0	QH118E_2 Goats: 5-9	-.014	-0.131434392	0.001576196
QH118E_3 Goats: 10+	.0107	.10291	5232	0	QH118E_3 Goats: 10+	-.023	-0.220073397	0.00238101
QH118F_1 Sheep: 1-4	.0273	.16306	5232	0	QH118F_1 Sheep: 1-4	-.025	-0.151178432	0.004248087

QH118F_2 Sheep: 5-9	.0097	.09826	5232	0
QH118F_3 Sheep: 10+	.0055	.07425	5232	0
QH118G_1 Chickens or other poultry: 1-9	.0761	.26514	5232	0
QH118G_2 Chickens or other poultry: 10-29	.0126	.11162	5232	0
QH118G_3 Chickens or other poultry: 30+	.0027	.05166	5232	0
QH118H_1 Beehives: 1-9	.0061	.07797	5232	0
QH118H_2 Beehives: 10-29	.0011	.03385	5232	0
QH118H_3 Beehives: 30+	.0002	.01383	5232	0
landarea	.2740	3.2117	5232	55

a. For each variable, missing values are replaced with the variable mean.

QH118F_2 Sheep: 5-9	-.018	-0.176536306	0.001737763
QH118F_3 Sheep: 10+	-.019	-0.259283741	0.001445172
QH118G_1 Chickens or other poultry: 1-9	-.035	-0.122496603	0.010085571
QH118G_2 Chickens or other poultry: 10-29	-.013	-0.116695647	0.001490885
QH118G_3 Chickens or other poultry: 30+	-.003	-0.062043192	0.000166463
QH118H_1 Beehives: 1-9	-.014	-0.173853804	0.00106987
QH118H_2 Beehives: 10-29	-.004	-0.115708942	0.000132846
QH118H_3 Beehives: 30+	-.004	-0.30925532	5.91197E-05
landarea	-.005	((landarea-2.7396)/3.21169)*(-0.00466)	

Extraction Method: Principal Component Analysis. Component Scores

Rural

Descriptive Statistics

	Mean	Std. Deviation[a]	Analysis N[a]	Missing N
QH101_11 Source of drinking water: Piped into dwelling	.00	.045	11418	0
QH101_12 Source of drinking water: Piped to yard/plot	.02	.123	11418	0
QH101_13 Source of drinking water: Piped to neighbor	.01	.101	11418	0
QH101_14 Source of drinking water: Public tap/standpipe	.16	.370	11418	0
QH101_21 Source of drinking water: Tube well or borehole	.19	.390	11418	0
QH101_31 Source of drinking water: Protected well	.08	.279	11418	0
QH101_32 Source of drinking water: Unprotected well	.08	.277	11418	0
QH101_41 Source of drinking water: Protected spring	.11	.308	11418	0
QH101_42 Source of drinking water: Unprotected spring	.18	.381	11418	0
QH101_51 Source of drinking water: Rainwater	.01	.086	11418	0
QH101_61 Source of drinking water: Tanker truck	.01	.078	11418	0
QH101_71 Source of drinking water: Cart with small tank	.00	.050	11418	0
QH101_81 Source of drinking water: Surface water (river/dam/lake/pond/stream/canal/irrigation channel)	.15	.360	11418	0
QH101_91 Source of drinking water: Bottled water	.00	.032	11418	0
QH101_96 Source of drinking water: Other	.00	.035	11418	0
QH109_12 Type of toilet facility: Flush to septic tank	.00	.037	11418	0
QH109_13 Type of toilet facility: Flush to pit latrine	.01	.094	11418	0
QH109_15 Type of toilet facility: Flush, don't know where	.00	.026	11418	0
QH109_21 Type of toilet facility: Ventilated improved pit latrine	.00	.034	11418	0
QH109_22 Type of toilet facility: Pit latrine with slab	.03	.161	11418	0
QH109_23 Type of toilet facility: Pit latrine without slab/open pit	.34	.474	11418	0
QH109_31 Type of toilet facility: Composting toilet	.01	.084	11418	0
QH109_41 Type of toilet facility: Bucket toilet	.00	.009	11418	0
QH109_51 Type of toilet facility: Hanging toilet/hanging latrine	.00	.016	11418	0
QH109_61 Type of toilet facility: No facility/bush/field	.48	.500	11418	0
QH109_96 Type of toilet facility: Other	.00	.034	11418	0
QH109_11_sh Type of toilet facility: Flush to piped sewer system - shared	.00	.009	11418	0
QH109_12_sh Type of toilet facility: Flush to septic tank - shared	.00	.016	11418	0
QH109_13_sh Type of toilet facility: Flush to pit latrine - shared	.00	.063	11418	0
QH109_14_sh Type of toilet facility: Flush to somewhere else shared	.00	.009	11418	0
QH109_15_sh Type of toilet facility: Flush, don't know where - shared	.00	.009	11418	0

Component Score Coefficient Matrix

	Component 1
QH101_11 Source of drinking water: Piped into dwelling	.023
QH101_12 Source of drinking water: Piped to yard/plot	.052
QH101_13 Source of drinking water: Piped to neighbor	.016
QH101_14 Source of drinking water: Public tap/standpipe	.021
QH101_21 Source of drinking water: Tube well or borehole	.014
QH101_31 Source of drinking water: Protected well	-.006
QH101_32 Source of drinking water: Unprotected well	-.055
QH101_41 Source of drinking water: Protected spring	.029
QH101_42 Source of drinking water: Unprotected spring	.002
QH101_51 Source of drinking water: Rainwater	-.015
QH101_61 Source of drinking water: Tanker truck	-.001
QH101_71 Source of drinking water: Cart with small tank	.000
QH101_81 Source of drinking water: Surface water (river/dam/lake/pond/stream/canal/irrigation channel)	-.039
QH101_91 Source of drinking water: Bottled water	.030
QH101_96 Source of drinking water: Other	-.008
QH109_12 Type of toilet facility: Flush to septic tank	.013
QH109_13 Type of toilet facility: Flush to pit latrine	.009
QH109_15 Type of toilet facility: Flush, don't know where	-.002
QH109_21 Type of toilet facility: Ventilated improved pit latrine	.028
QH109_22 Type of toilet facility: Pit latrine with slab	.031
QH109_23 Type of toilet facility: Pit latrine without slab/open pit	.077
QH109_31 Type of toilet facility: Composting toilet	.004
QH109_41 Type of toilet facility: Bucket toilet	.009
QH109_51 Type of toilet facility: Hanging toilet/hanging latrine	-.001
QH109_61 Type of toilet facility: No facility/bush/field	-.114
QH109_96 Type of toilet facility: Other	.002
QH109_11_sh Type of toilet facility: Flush to piped sewer system - shared	.003
QH109_12_sh Type of toilet facility: Flush to septic tank - shared	.004
QH109_13_sh Type of toilet facility: Flush to pit latrine - shared	.000
QH109_14_sh Type of toilet facility: Flush to somewhere else - shared	.001
QH109_15_sh Type of toilet facility: Flush, don't know where - shared	-.002

Sum over each variable

If has	If does not have
0.507972122	-0.001025306
0.417369356	-0.006458757
0.156748318	-0.001636841
0.047444944	-0.009292213
0.028374145	-0.006518261
-0.02111861	0.00195625
-0.18304668	0.016688316
0.085289275	-0.010091033
0.004532948	-0.00097138
-0.17695474	0.001342932
-0.01291344	7.85115E-05
0.002535564	-6.45635E-06
-0.09227491	0.016646307
0.922107826	-0.000970129
-0.22499806	0.000276216
0.351324314	-0.000493
0.091518124	-0.000824925
-0.06247094	4.38008E-05
0.822421282	-0.000937438
0.188801236	-0.005164259
0.107144271	-0.055473473
0.041794991	-0.000302328
0.975992101	-8.54859E-05
-0.05138779	1.35053E-05
-0.11812568	0.10966676
0.068729701	-7.83416E-05
0.300217329	-2.62956E-05
0.244001661	-6.41266E-05
0.002522537	-9.98102E-06
0.05783764	-5.06592E-06
-0.17029299	1.49157E-05

QH109_21_sh Type of toilet facility: Ventilated improved pit latrine - shared	.00	.034	11418	0	QH109_21_sh Type of toilet facility: Ventilated improved pit latrine - shared	.004	0.103714374	-0.000118219
QH109_22_sh Type of toilet facility: Pit latrine with slab - shared	.02	.156	11418	0	QH109_22_sh Type of toilet facility: Pit latrine with slab - shared	.023	0.144235044	-0.003692355
QH109_23_sh Type of toilet facility: Pit latrine without slab/open pit - shared	.10	.294	11418	0	QH109_23_sh Type of toilet facility: Pit latrine without slab/open pit - shared	.030	0.092156556	-0.00972605
QH109_31_sh Type of toilet facility: Composting toilet - shared	.00	.036	11418	0	QH109_31_sh Type of toilet facility: Composting toilet - shared	.003	0.079512682	-0.000104594
QH109_96_sh Type of toilet facility: Other - shared	.00	.051	11418	0	QH109_96_sh Type of toilet facility: Other - shared	-.001	-0.02692562	7.09316E-05
QH113_1 Type of cooking fuel: Electricity	.00	.064	11418	0	QH113_1 Type of cooking fuel: Electricity	.036	0.56077714	-0.002317872
QH113_2 Type of cooking fuel: LPG	.00	.046	11418	0	QH113_2 Type of cooking fuel: LPG	.008	0.173492478	-0.00036544
QH113_3 Type of cooking fuel: Natural gas	.00	.013	11418	0	QH113_3 Type of cooking fuel: Natural gas	.003	0.23040271	-4.03649E-05
QH113_4 Type of cooking fuel: Biogas	.00	.013	11418	0	QH113_4 Type of cooking fuel: Biogas	.003	0.236160829	-4.13737E-05
QH113_5 Type of cooking fuel: Kerosene	.00	.026	11418	0	QH113_5 Type of cooking fuel: Kerosene	.014	0.53727057	-0.000376702
QH113_6 Type of cooking fuel: Charcoal	.03	.170	11418	0	QH113_6 Type of cooking fuel: Charcoal	.040	0.225144463	-0.006951915
QH113_7 Type of cooking fuel: Wood	.89	.312	11418	0	QH113_7 Type of cooking fuel: Wood	-.059	-0.02061194	0.168118743
QH113_8 Type of cooking fuel: Straw/shrubs/grass	.00	.063	11418	0	QH113_8 Type of cooking fuel: Straw/shrubs/grass	.005	0.085589228	-0.000338654
QH113_9 Type of cooking fuel: Agricultural crop	.01	.109	11418	0	QH113_9 Type of cooking fuel: Agricultural crop	.013	0.118168044	-0.001445673
QH113_10 Type of cooking fuel: Animal dung	.05	.215	11418	0	QH113_10 Type of cooking fuel: Animal dung	.029	0.127494877	-0.0065385
QH113_95 Type of cooking fuel: No food cooked in household	.01	.084	11418	0	QH113_95 Type of cooking fuel: No food cooked in household	.006	0.06921459	-0.000494521
QH113_96 Type of cooking fuel: Other	.00	.009	11418	0	QH113_96 Type of cooking fuel: Other	.001	0.113295696	-9.92342E-06
QH121A Electricity	.10	.305	11418	0	QH121A Electricity	.085	0.248638267	-0.028819877
QH121B Radio	.22	.412	11418	0	QH121B Radio	.065	0.12311594	-0.034125425
QH121C Television	.03	.169	11418	0	QH121C Television	.078	0.448384494	-0.013678155
QH121D Telephone (non-mobile)	.01	.072	11418	0	QH121D Telephone (non-mobile)	.031	0.425513992	-0.002247829
QH121E Computer	.00	.048	11418	0	QH121E Computer	.028	0.592942871	-0.001353276
QH121F Refrigerator	.01	.084	11418	0	QH121F Refrigerator	.055	0.644520821	-0.004662201
QH121G Table	.25	.431	11418	0	QH121G Table	.096	0.167338057	-0.054522827
QH121H Chair	.34	.475	11418	0	QH121H Chair	.086	0.118612508	-0.061770765
QH121I Bed with cotton/spring mattress	.23	.419	11418	0	QH121I Bed with cotton/spring mattress	.093	0.171230663	-0.050236454
QH121J Electric mitad	.01	.078	11418	0	QH121J Electric mitad	.054	0.687706584	-0.00418114
QH121K Kerosene lamp/ preassure lamp	.09	.292	11418	0	QH121K Kerosene lamp/ preassure lamp	.012	0.038264146	-0.003968819
QH122A Watch	.22	.417	11418	0	QH122A Watch	.014	0.025866056	-0.007456592
QH122B Mobile telephone	.48	.500	11418	0	QH122B Mobile telephone	.061	0.063417943	-0.058855963
QH122C Bicycle	.01	.117	11418	0	QH122C Bicycle	.031	0.26176136	-0.003673028
QH122D Motorcycle or scooter	.01	.084	11418	0	QH122D Motorcycle or scooter	.020	0.2380154	-0.001721706
QH122E Animal-drawn cart	.01	.121	11418	0	QH122E Animal-drawn cart	.009	0.072456227	-0.00108855
QH122F Car or Truck	.00	.047	11418	0	QH122F Car or Truck	.026	0.552214086	-0.00121174
QH122G Boat with a motor	.00	.037	11418	0	QH122G Boat with a motor	.013	0.335736804	-0.000471127
QH122H Bagag	.00	.056	11418	0	QH122H Bagag	.019	0.343496261	-0.00108644
QH123 Bank account	.16	.370	11418	0	QH123 Bank account	.079	0.179039728	-0.034908531
QH142_11 Main floor material: Earth/sand	.68	.465	11418	0	QH142_11 Main floor material: Earth/sand	-.093	-0.06305126	0.135821472
QH142_12 Main floor material: Dung	.24	.428	11418	0	QH142_12 Main floor material: Dung	.053	0.094502361	-0.030197507
QH142_21 Main floor material: Wood planks	.00	.030	11418	0	QH142_21 Main floor material: Wood planks	.000	-0.01521296	1.33353E-05
QH142_22 Main floor material: Palm/bamboo	.01	.095	11418	0	QH142_22 Main floor material: Palm/bamboo	.024	0.25053271	-0.002280589
QH142_31 Main floor material: Parquet or polished wood	.00	.019	11418	0	QH142_31 Main floor material: Parquet or polished wood	.007	0.382315843	-0.000133981
QH142_32 Main floor material: Vinyl or asphalt strips	.02	.131	11418	0	QH142_32 Main floor material: Vinyl or asphalt strips	.032	0.238894026	-0.004237446
QH142_33 Main floor material: Ceramic tiles	.00	.030	11418	0	QH142_33 Main floor material: Ceramic tiles	.017	0.57246839	-0.000501813
QH142_34 Main floor material: Cement	.02	.150	11418	0	QH142_34 Main floor material: Cement	.048	0.313080076	-0.007352723
QH142_35 Main floor material: Carpet	.02	.149	11418	0	QH142_35 Main floor material: Carpet	.040	0.259603641	-0.006049198

QH142_96 Main floor material: Other	.00	.025	11418	0	QH142_96 Main floor material: Other	.001	0.057739426	-3.54199E-05
QH143_11 Main roof material: No roof	.00	.046	11418	0	QH143_11 Main roof material: No roof	-.007	-0.16205482	0.000341348
QH143_12 Main roof material: Thatch/mud	.08	.274	11418	0	QH143_12 Main roof material: Thatch/mud	-.021	-0.06961927	0.00619502
QH143_13 Main roof material: Sod	.34	.473	11418	0	QH143_13 Main roof material: Sod	-.033	-0.04574646	0.023272184
QH143_21 Main roof material: Rustic mat	.09	.284	11418	0	QH143_21 Main roof material: Rustic mat	-.102	-0.32749915	0.03191889
QH143_22 Main roof material: Palm/bamboo	.01	.105	11418	0	QH143_22 Main roof material: Palm/bamboo	.000	0.002702091	-3.03928E-05
QH143_23 Main roof material: Wood planks	.02	.148	11418	0	QH143_23 Main roof material: Wood planks	-.019	-0.12754827	0.002925314
QH143_24 Main roof material: Cardboard	.00	.040	11418	0	QH143_24 Main roof material: Cardboard	-.012	-0.30169591	0.000476362
QH143_31 Main roof material: Metal/ corrugated iron	.42	.494	11418	0	QH143_31 Main roof material: Metal/ corrugated iron	.115	0.134095598	-0.09887816
QH143_32 Main roof material: Wood	.01	.078	11418	0	QH143_32 Main roof material: Wood	-.004	-0.05098773	0.000309997
QH143_33 Main roof material: Calamine/cement fiber	.00	.009	11418	0	QH143_33 Main roof material: Calamine/cement fiber	.002	0.196109911	-1.7177E-05
QH143_35 Main roof material: Cement	.02	.137	11418	0	QH143_35 Main roof material: Cement	-.007	-0.04791574	0.000932646
QH143_36 Main roof material: Roofing shingles	.00	.013	11418	0	QH143_36 Main roof material: Roofing shingles	-.003	-0.22901899	4.01225E-05
QH143_96 Main roof material: Other	.01	.072	11418	0	QH143_96 Main roof material: Other	-.025	-0.34682861	0.001832164
QH144_11 Main wall material: No walls	.01	.107	11418	0	QH144_11 Main wall material: No walls	-.040	-0.37392831	0.004373431
QH144_12 Main wall material: Cane/palm/trunks	.12	.319	11418	0	QH144_12 Main wall material: Cane/palm/trunks	-.072	-0.19873637	0.025889632
QH144_13 Main wall material: Dirt	.02	.149	11418	0	QH144_13 Main wall material: Dirt	-.056	-0.36639047	0.008470317
QH144_21 Main wall material: Bamboo with mud	.66	.473	11418	0	QH144_21 Main wall material: Bamboo with mud	.077	0.05459555	-0.107656399
QH144_22 Main wall material: Stone with mud	.13	.334	11418	0	QH144_22 Main wall material: Stone with mud	.005	0.014304852	-0.00209567
QH144_23 Main wall material: Uncovered adobe	.00	.026	11418	0	QH144_23 Main wall material: Uncovered adobe	.004	0.13237806	-9.28155E-05
QH144_24 Main wall material: Plywood	.00	.040	11418	0	QH144_24 Main wall material: Plywood	.002	0.043281442	-6.83391E-05
QH144_25 Main wall material: Cardboard	.00	.030	11418	0	QH144_25 Main wall material: Cardboard	-.006	-0.2138265	0.000187436
QH144_26 Main wall material: Reused wood	.01	.095	11418	0	QH144_26 Main wall material: Reused wood	-.021	-0.22388134	0.002037983
QH144_31 Main wall material: Cement	.01	.099	11418	0	QH144_31 Main wall material: Cement	.025	0.251680747	-0.002538182
QH144_32 Main wall material: Stone with lime/cement	.01	.107	11418	0	QH144_32 Main wall material: Stone with lime/cement	.028	0.259062226	-0.003053192
QH144_33 Main wall material: Bricks	.00	.013	11418	0	QH144_33 Main wall material: Bricks	.009	0.660291476	-0.000115678
QH144_34 Main wall material: Cement blocks	.00	.031	11418	0	QH144_34 Main wall material: Cement blocks	.014	0.443579935	-0.000427753
QH144_35 Main wall material: Covered adobe	.00	.031	11418	0	QH144_35 Main wall material: Covered adobe	-.006	-0.17919982	0.000172806
QH144_36 Main wall material: Wood planks/shingles	.01	.088	11418	0	QH144_36 Main wall material: Wood planks/shingles	-.020	-0.2288759	0.001798036
QH144_96 Main wall material: Other	.02	.124	11418	0	QH144_96 Main wall material: Other	-.037	-0.2961127	0.004689329
DOMESTIC Domestic staff	.00	.025	11418	0	DOMESTIC Domestic staff	.009	0.349537635	-0.000214421
HOUSE Owns a house	.67	.472	11418	0	HOUSE Owns a house	.007	0.005267645	-0.010460904
LAND Owns land	.83	.371	11418	0	LAND Owns land	.038	0.017125764	-0.086500084
memsleep Number of members per sleeping room	3.98	2.210	11418	6	memsleep Number of members per sleeping room	-.04183	((memsleep-3.98107)/2.20953)*(-0.04183)	
QH118A_1 Cows/bulls: 1-4	.4723	.49926	11418	0	QH118A_1 Cows/bulls: 1-4	.035	0.037298352	-0.033385894
QH118A_2 Cows/bulls: 5-9	.0916	.28849	11418	0	QH118A_2 Cows/bulls: 5-9	-.001	-0.00177712	0.000179219
QH118A_3 Cows/bulls: 10+	.0432	.20327	11418	0	QH118A_3 Cows/bulls: 10+	-.031	-0.14727512	0.006645916
QH118B_1 Other cattle: 1-4	.3205	.46667	11418	0	QH118B_1 Other cattle: 1-4	.029	0.042407112	-0.019998405
QH118B_2 Other cattle: 5-9	.0281	.16530	11418	0	QH118B_2 Other cattle: 5-9	-.013	-0.07924286	0.002292237
QH118B_3 Other cattle: 10+	.0090	.09455	11418	0	QH118B_3 Other cattle: 10+	-.014	-0.14248109	0.001297
QH118C_1 Horses/donkeys/mules: 1-4	.3531	.47796	11418	0	QH118C_1 Horses/donkeys/mules: 1-4	-.010	-0.01371489	0.007486926
QH118C_2 Horses/donkeys/mules: 5-9	.0034	.05835	11418	0	QH118C_2 Horses/donkeys/mules: 5-9	.001	0.020294633	-6.95571E-05
QH118C_3 Horses/donkeys/mules: 10+	.0004	.02092	11418	0	QH118C_3 Horses/donkeys/mules: 10+	-.007	-0.32936117	0.000144292
QH118D_1 Camels: 1-4	.0474	.21246	11418	0	QH118D_1 Camels: 1-4	-.058	-0.26147611	0.013005293
QH118D_2 Camels: 5-9	.0131	.11387	11418	0	QH118D_2 Camels: 5-9	-.040	-0.34926709	0.004649455
QH118D_3 Camels: 10+	.0111	.10488	11418	0	QH118D_3 Camels: 10+	-.035	-0.32651678	0.003672627
QH118E_1 Goats: 1-4	.1803	.38448	11418	0	QH118E_1 Goats: 1-4	.013	0.027188713	-0.005981575
QH118E_2 Goats: 5-9	.0719	.25834	11418	0	QH118E_2 Goats: 5-9	-.009	-0.0337291	0.002613154
QH118E_3 Goats: 10+	.1364	.34319	11418	0	QH118E_3 Goats: 10+	-.103	-0.2598518	0.041029232
QH118F_1 Sheep: 1-4	.1831	.38679	11418	0	QH118F_1 Sheep: 1-4	.020	0.042992701	-0.009638441
QH118F_2 Sheep: 5-9	.0678	.25139	11418	0	QH118F_2 Sheep: 5-9	-.007	-0.02771988	0.002015707
QH118F_3 Sheep: 10+	.0650	.24651	11418	0	QH118F_3 Sheep: 10+	-.067	-0.253858	0.017643559

QH118G_1 Chickens or other poultry: 1-9	.3934	.48853	11418	0
QH118G_2 Chickens or other poultry: 10-29	.0714	.25747	11418	0
QH118G_3 Chickens or other poultry: 30+	.0043	.06537	11418	0
QH118H_1 Beehives: 1-9	.0916	.28849	11418	0
QH118H_2 Beehives: 10-29	.0135	.11535	11418	0
QH118H_3 Beehives: 30+	.0053	.07230	11418	0
landarea	1.2403	3.74796	11418	169

a. For each variable, missing values are replaced with the variable mean.

QH118G_1 Chickens or other poultry: 1-9	.048
QH118G_2 Chickens or other poultry: 10-29	.014
QH118G_3 Chickens or other poultry: 30+	-.001
QH118H_1 Beehives: 1-9	.026
QH118H_2 Beehives: 10-29	.003
QH118H_3 Beehives: 30+	.002
landarea	.00270

Extraction Method: Principal Component Analysis.

[illegible]

0.059657182	-0.038691895
0.050374989	-0.003872075
-0.01470042	6.33583E-05
0.080989504	-0.008167665
0.025470138	-0.000348225
0.031747917	-0.000167712

((landarea-1.24028)/3.74796)*(-0.00270.)

Combined Scores

Urban Area

Coefficients[a]

Model		Unstandardized Coefficients B	Unstandardized Coefficients Std. Error	Standardized Coefficients Beta	t	Sig.
1	(Constant)	1.206	.002		560.438	0.000
	urbscore Urban wealth score	.816	.002	.982	379.471	0.000

a. Dependent Variable: comscore Common wealth score

Combined Score= 1.206 +.002 * Urban Score

Rural

Coefficients[a]

Model		Unstandardized Coefficients B	Unstandardized Coefficients Std. Error	Standardized Coefficients Beta	t	Sig.
1	(Constant)	-.552	.002		-239.740	0.000
	rurscore Rural wealth score	.332	.002	.803	143.910	0.000

a. Dependent Variable: comscore Common wealth score

Combined Score= -552+ .332 * Rural Score

Combined Score

Statistics

combscor Combined national wealth score

N	Valid	16650
	Missing	0
Mean		-.1395789
Std. Error of Mean		.00579978
Median		-.3860525
Mode		.74793
Std. Deviation		.74837441
Skewness		1.828
Std. Error of Skewness		.019
Kurtosis		2.795
Std. Error of Kurtosis		.038
Minimum		-2.13343
Maximum		3.09962
Percentiles	20	-.6093627
	40	-.4614207
	60	-.3050082
	80	.0604099

histrogram

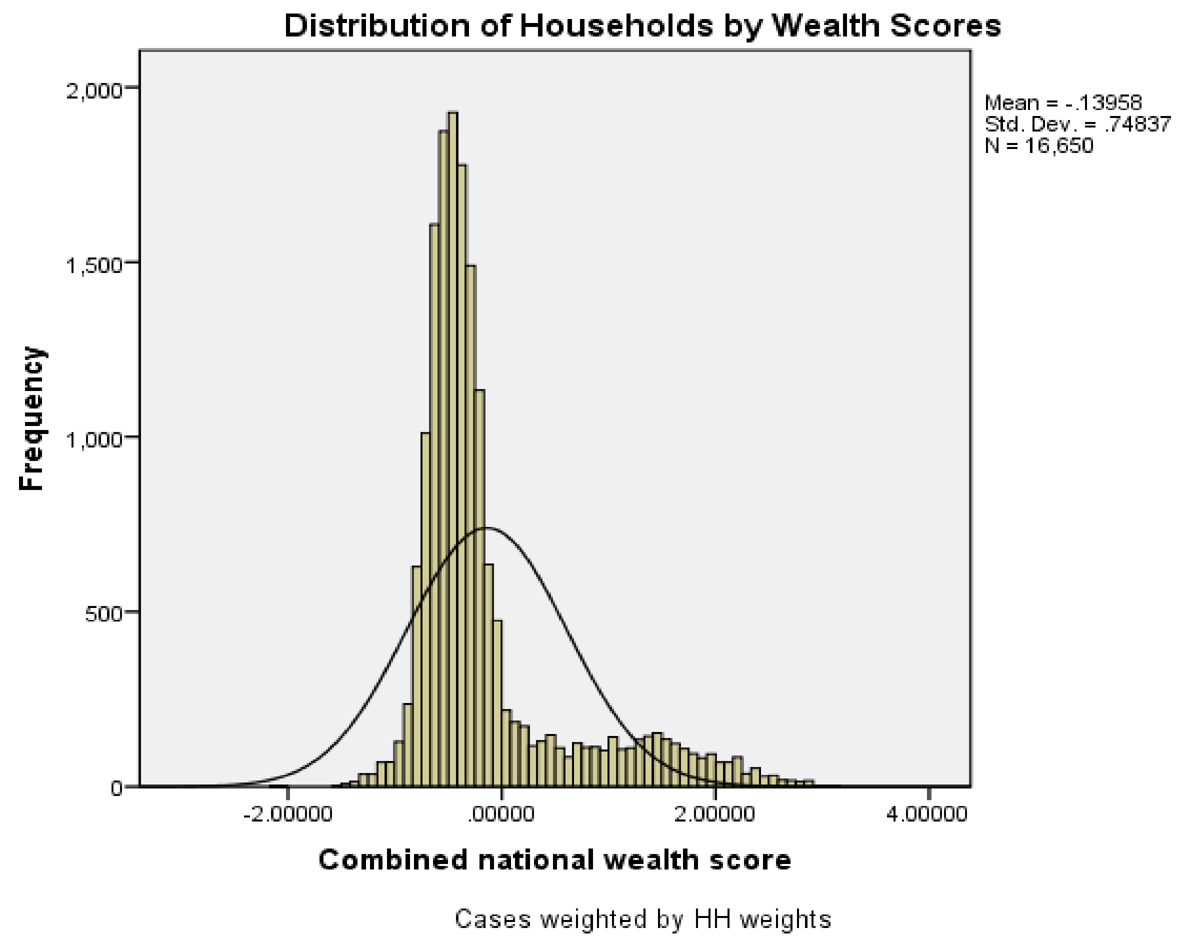

	Ncombsco Combined wealth index					Nurbscor Urban wealth index					Nrurscor Rural wealth index				
	Lowest	Second	Middle	Fourth	Highest	Lowest	Second	Middle	Fourth	Highest	Lowest	Second	Middle	Fourth	Highest
QH101_11 Source of drinking water: Piped - into dwelling	.030	.058	.085	.097	.103	.076	.114	.118	.115	.111	.015	.038	.044	.052	.062
QH101_12 Source of drinking water: Piped - to yard/plot	.013	.012	.011	.010	.009	.019	.012	.013	.010	.009	.008	.013	.009	.009	.007
QH101_13 Source of drinking water: Piped - to neighbor	.016	.010	.006	.002	.000	.029	.008	.005	.001	0.000	.008	.008	.005	.003	.001

QH101_14 Source of drinking water: Piped - public tap / standpipe	.005	.008	.006	.006	.003	.009	.008	.008	.004	.002	.005	.005	.006	.003	.004
QH101_21 Source of drinking water: Tube well or borehole	.117	.166	.195	.196	.148	.148	.182	.181	.194	.113	.087	.159	.179	.207	.194
QH101_31 Source of drinking water: Dug well - protected	.206	.239	.180	.137	.063	.210	.142	.120	.071	.034	.189	.236	.261	.222	.166
QH101_32 Source of drinking water: Dug well - unprotected	.116	.056	.036	.016	.005	.049	.021	.009	.006	.002	.139	.100	.060	.048	.025
QH101_41 Source of drinking water: Spring protected	.194	.123	.080	.046	.016	.075	.041	.024	.018	.003	.229	.188	.147	.124	.071
QH101_42 Source of drinking water: Spring unprotected	.079	.022	.010	.005	.001	.021	.002	.003	.000	.000	.111	.042	.029	.017	.007
QH101_51 Source of drinking water: Rainwater	.049	.027	.016	.010	.002	.016	.010	.008	.004	.001	.063	.040	.033	.022	.011
QH101_61 Source of drinking water: Tanker truck	.007	.007	.006	.001	.001	.003	.001	.000	.001	.001	.009	.008	.008	.010	.002
QH101_71 Source of drinking water: Cart with small tank	.005	.009	.009	.013	.008	.007	.009	.015	.010	.006	.002	.008	.010	.008	.010

QH101_81 Source of drinking water: Surface water (river/dam/lake/pond/stream/canal/irrigation channel)	.061	.012	.004	.001	.000	.009	.001	0.000	.001	0.000	.092	.034	.011	.008	.001
QH101_91 Source of drinking water: Bottled water	.007	.030	.068	.133	.391	.044	.102	.150	.264	.500	.001	.006	.016	.031	.146
QH101_92 Source of drinking water: Refilled water	.097	.222	.289	.326	.249	.286	.346	.346	.302	.217	.041	.116	.181	.235	.292
QH101_96 Source of drinking water: Other	.000	.000	0.000	.000	.000	.000	0.000	0.000	0.000	.000	0.000	0.000	.000	0.000	.000
QH109_11 Type of toilet facility: Private with septic tank	.299	.640	.812	.925	.978	.473	.777	.907	.959	.989	.203	.502	.706	.849	.950
QH109_12 Type of toilet facility: Private with non septic tank	.113	.118	.098	.056	.019	.129	.111	.064	.034	.010	.105	.118	.112	.082	.042
QH109_21 Type of toilet facility: Shared/public	.178	.100	.041	.008	.001	.208	.064	.013	.002	.001	.180	.102	.059	.020	.004
QH109_31 Type of toilet facility: River/stream/creek	.197	.089	.037	.009	.002	.127	.042	.016	.005	0.000	.220	.149	.074	.033	.002
QH109_32 Type of toilet facility: Beach	.008	.002	.000	.000	0.000	.004	.000	0.000	0.000	0.000	.009	.006	.001	.000	.000

QH109_33 Type of toilet facility: Pool/ponds	.015	.006	.003	.000	.000	.009	.001	.000	0.000	0.000	.013	.014	.006	.004	.000
QH109_41 Type of toilet facility: Pit	.120	.036	.007	.001	.000	.031	.004	.001	.000	0.000	.158	.089	.034	.010	.001
QH109_51 Type of toilet facility: Yard/bush/forest	.069	.009	.001	0.000	0.000	.018	.001	0.000	0.000	0.000	.110	.021	.009	.001	0.000
QH109_96 Type of toilet facility: Other	.002	.000	.000	0.000	0.000	.001	.000	0.000	0.000	0.000	.002	.001	.000	.001	0.000
QH112B Distance between the well and the nearest septic tank	28.619	18.826	16.799	15.992	14.900	21.042	15.555	13.345	14.440	13.889	31.638	24.269	18.528	17.584	18.577
QH113_1 Type of cooking fuel: Electricity	.003	.006	.005	.003	.002	.011	.010	.004	.001	.002	.001	.002	.002	.002	.002
QH113_2 Type of cooking fuel: LPG	.240	.655	.855	.940	.973	.564	.857	.937	.965	.979	.118	.429	.678	.852	.947
QH113_3 Type of cooking fuel: Natural gas	.001	.002	.004	.005	.008	.002	.005	.006	.009	.010	.000	.001	.001	.003	.002
QH113_4 Type of cooking fuel: Biogas	.000	0.000	.000	.000	.000	0.000	0.000	.000	.000	.000	.000	.001	0.000	.000	0.000
QH113_5 Type of cooking fuel: Kerosene	.033	.045	.036	.028	.013	.078	.044	.030	.020	.007	.014	.030	.031	.028	.026
QH113_6 Type of cooking fuel: Coal, lignite	.000	0.000	.000	0.000	0.000	.000	0.000	0.000	0.000	0.000	.000	.000	0.000	.000	0.000
QH113_7 Type of cooking fuel: Charcoal	.003	.001	.001	.000	0.000	.002	.001	.000	0.000	0.000	.002	.003	.001	.001	.000

QH113_8 Type of cooking fuel: Wood	.699	.269	.079	.016	.002	.285	.044	.007	.002	0.000	.853	.528	.282	.113	.022
QH113_9 Type of cooking fuel: Straw/shrubs/grass/crops	.000	.000	0.000	.000	.000	.000	0.000	.000	0.000	.000	.001	.000	.000	0.000	0.000
QH113_10 Type of cooking fuel: Agricultural crop	.000	0.000	0.000	0.000	0.000	0.000	0.000	0.000	0.000	0.000	.000	0.000	0.000	0.000	0.000
QH113_11 Type of cooking fuel: Animal dung	0.000	0.000	0.000	0.000	0.000	0.000	0.000	0.000	0.000	0.000	0.000	0.000	0.000	0.000	0.000
QH113_95 Type of cooking fuel: No food cooked in household	.020	.021	.020	.007	.001	.056	.039	.014	.002	.001	.011	.006	.004	.003	.001
QH113_96 Type of cooking fuel: Other	0.000	0.000	0.000	0.000	0.000	0.000	0.000	0.000	0.000	0.000	0.000	0.000	0.000	0.000	0.000
QH121A Electricity	.891	.986	.994	.998	1.000	.957	.991	.997	.999	1.000	.833	.977	.988	.996	.999
QH121B Radio	.115	.174	.208	.283	.397	.161	.211	.273	.337	.441	.095	.148	.179	.202	.310
QH121C Television	.598	.925	.973	.990	.997	.794	.955	.986	.993	.999	.433	.874	.952	.986	.996
QH121D Non-mobile telephone	.001	.003	.005	.013	.153	.002	.007	.017	.043	.267	.001	.001	.002	.001	.010
QH121E Computer	.014	.050	.115	.272	.724	.044	.116	.213	.465	.868	.008	.027	.048	.119	.453
QH121F Refrigerator	.074	.341	.654	.899	.990	.220	.610	.850	.976	.996	.028	.184	.408	.707	.950
QH121G Fan	.170	.501	.712	.868	.951	.417	.703	.851	.918	.960	.073	.323	.526	.727	.910
QH121H Washing machine	.010	.079	.230	.492	.847	.050	.207	.419	.701	.916	.003	.031	.095	.259	.647
QH121I Air Conditioner	.000	.002	.008	.037	.398	.001	.010	.036	.150	.607	.000	.000	.002	.006	.088
QH122A Watch	.153	.325	.455	.653	.905	.260	.462	.612	.813	.950	.113	.239	.340	.451	.747
QH122B Mobile telephone	.631	.899	.969	.994	.999	.778	.960	.992	.999	1.000	.547	.811	.926	.977	.998

QH122C Bicycle	.170	.314	.401	.480	.651	.258	.359	.430	.535	.701	.115	.248	.350	.452	.591
QH122D Motorcycle or scooter	.447	.762	.879	.946	.966	.542	.822	.920	.963	.954	.342	.699	.848	.937	.982
QH122E Animal-drawn cart	.002	.002	.001	.002	.003	.002	.001	.001	.001	.003	.001	.002	.002	.001	.004
QH122F Car or Truck	.003	.014	.040	.108	.513	.006	.025	.061	.171	.691	.002	.007	.020	.063	.321
QH122G Boat with a motor	.016	.009	.006	.004	.005	.004	.003	.003	.002	.005	.019	.017	.009	.008	.008
QH123 Bank account	.144	.333	.516	.739	.958	.242	.507	.683	.894	.985	.116	.227	.371	.527	.837
QH142_11 Main floor material: Earth/sand/dung	.157	.053	.013	.003	.001	.077	.010	.002	.001	.000	.189	.104	.050	.017	.004
QH142_12 Main floor material: Dung	.000	0.000	0.000	0.000	0.000	0.000	0.000	0.000	0.000	0.000	.000	.001	0.000	0.000	0.000
QH142_21 Main floor material: Wood planks	.243	.112	.055	.024	.003	.126	.054	.025	.007	.001	.301	.173	.109	.055	.020
QH142_22 Main floor material: Palm/bamboo	.024	.002	.000	0.000	0.000	.006	.000	0.000	0.000	0.000	.040	.004	.001	0.000	0.000
QH142_31 Main floor material: Parquet or polished wood	.002	.001	.002	.001	.000	.001	.001	.001	.001	0.000	.002	.001	.001	.003	.001
QH142_32 Main floor material: Vinyl or asphalt strips	.001	.002	.001	.001	.002	.001	.001	.001	.001	.002	.001	.001	.003	.001	.001
QH142_33 Main floor material: Ceramic/Marble	.080	.315	.554	.800	.951	.217	.557	.783	.918	.973	.036	.182	.359	.556	.828

QH142_34 Main floor material: Ceramic tiles	.048	.081	.079	.056	.027	.103	.088	.066	.040	.020	.029	.058	.068	.068	.040
QH142_35 Main floor material: Cement/Red bricks	.439	.428	.291	.111	.016	.456	.285	.117	.032	.002	.396	.470	.407	.296	.104
QH142_36 Main floor material: Carpet	.004	.006	.002	.002	.000	.009	.003	.003	.000	0.000	.003	.005	.002	.002	.001
QH142_96 Main floor material: Other	.001	.001	.000	.000	0.000	.001	.001	.000	0.000	0.000	.001	.001	.000	0.000	0.000
QH142A the floor area of the house	58.871	70.042	78.223	87.163	131.795	59.170	69.039	78.372	95.873	145.599	53.478	70.012	76.842	87.756	117.578
QH143_12 Main roof material: Thatch/palm leaf	.038	.002	.000	.000	0.000	.008	.000	.000	0.000	0.000	.064	.008	.001	.000	0.000
QH143_13 Main roof material: Sod	.006	0.000	.000	0.000	0.000	.000	.000	0.000	0.000	0.000	.011	0.000	0.000	0.000	0.000
QH143_21 Main roof material: Rustic mat	.001	.000	.000	0.000	0.000	.001	0.000	0.000	0.000	0.000	.001	.000	.001	.000	0.000
QH143_22 Main roof material: Palm/bamboo	.001	.000	0.000	0.000	0.000	.000	0.000	0.000	0.000	0.000	.001	0.000	0.000	0.000	0.000
QH143_23 Main roof material: Wood planks	.001	.000	.000	0.000	.000	.001	.000	0.000	.000	0.000	.001	.000	.000	0.000	0.000
QH143_31 Main roof material: Roofing	.427	.328	.268	.206	.135	.320	.262	.200	.163	.109	.483	.382	.323	.274	.209
QH143_32 Main roof material: Asbestos	.067	.080	.108	.138	.097	.124	.167	.193	.166	.089	.056	.058	.049	.040	.032

QH143_33 Main roof material: Tile	.447	.572	.599	.625	.682	.532	.546	.579	.626	.692	.371	.533	.608	.663	.704
QH143_34 Main roof material: Concrete	.003	.006	.009	.015	.029	.007	.013	.018	.020	.034	.002	.005	.003	.005	.016
QH143_35 Main roof material: Metal tile	.003	.006	.011	.014	.055	.002	.008	.008	.022	.073	.002	.006	.008	.013	.035
QH143_36 Main roof material: Roofing shingles	.006	.005	.003	.002	.001	.003	.003	.001	.001	.001	.006	.008	.005	.003	.003
QH143_96 Main roof material: Other	.001	.000	.000	.000	.000	0.000	.000	.000	.000	.001	.001	0.000	.000	0.000	0.000
QH144_12 Main wall material: Cane/palm/trunks	.039	.005	.001	.000	0.000	.015	.002	.000	0.000	0.000	.059	.012	.003	.001	.000
QH144_13 Main wall material: Dirt	.000	0.000	0.000	0.000	0.000	0.000	0.000	0.000	0.000	0.000	.000	0.000	0.000	0.000	0.000
QH144_21 Main wall material: Bamboo with mud/Dirt	.007	.004	.001	.000	0.000	.006	.001	0.000	0.000	0.000	.007	.006	.004	.001	0.000
QH144_22 Main wall material: Stone with mud	.001	.000	.000	.000	0.000	.000	.000	0.000	0.000	0.000	.000	.001	.000	.000	.000
QH144_23 Main wall material: Uncovered adobe	.072	.064	.035	.010	.002	.076	.032	.011	.003	.002	.064	.073	.060	.035	.010
QH144_24 Main wall material: Plywood	.023	.013	.009	.002	.000	.026	.012	.003	.001	.000	.024	.013	.008	.006	.000

QH144_25 Main wall material: Cardboard	.000	.000	0.000	.000	0.000	.000	0.000	0.000	0.000	0.000	.000	.000	.000	0.000	.000
QH144_26 Main wall material: Reused wood	.005	.002	.001	.000	0.000	.006	.001	.000	0.000	0.000	.003	.002	.001	.000	0.000
QH144_31 Main wall material: Woven Bamboo	.120	.033	.008	.001	.001	.076	.008	.001	.000	0.000	.139	.064	.027	.008	.002
QH144_32 Main wall material: Stone with lime/cement	.023	.024	.025	.021	.018	.022	.029	.022	.022	.016	.022	.024	.025	.021	.019
QH144_34 Main wall material: Cement blocks	.056	.071	.082	.081	.061	.083	.107	.098	.083	.059	.048	.060	.059	.055	.047
QH144_35 Main wall material: Covered adobe	.223	.548	.718	.839	.909	.453	.705	.825	.880	.919	.125	.384	.580	.735	.873
QH144_36 Main wall material: Wood planks/shingles	.401	.209	.108	.041	.007	.205	.092	.036	.010	.002	.476	.337	.208	.121	.045
QH144_37 Main wall material: Plaster wire	.004	.006	.005	.003	.001	.003	.003	.002	.001	.001	.003	.005	.008	.007	.003
QH144_38 Main wall material: GRC/Gypsum/Asbestos	.025	.019	.008	.002	.000	.028	.007	.001	.000	.001	.026	.019	.015	.009	.001
QH144_96 Main wall material: Other	.001	.002	.001	.000	.000	.001	.000	.001	.000	.000	.002	.001	.002	.001	0.000

DOMESTIC Domestic staff	0.000	0.000	0.000	0.000	0.000	0.000	0.000	0.000	0.000	0.000	0.000	0.000	0.000	0.000	0.000
HOUSE Owns a house	.428	.480	.499	.555	.650	.333	.405	.470	.565	.665	.443	.501	.551	.603	.698
LAND Owns land	.536	.488	.461	.458	.519	.260	.294	.323	.379	.500	.618	.593	.604	.637	.731
memsleep Number of members per sleeping room	1.937	1.718	1.640	1.560	1.301	1.964	1.840	1.734	1.562	1.214	2.029	1.712	1.568	1.423	1.245
QH118A_1 Cows/bulls: 1-4	.078	.084	.062	.041	.015	.033	.023	.016	.010	.004	.075	.111	.111	.105	.073
QH118A_2 Cows/bulls: 5-9	.005	.006	.005	.003	.002	.001	.001	.001	.000	.000	.005	.007	.009	.009	.009
QH118A_3 Cows/bulls: 10+	.000	.001	.001	.002	.002	.000	.000	.001	.000	.001	.000	.001	.001	.002	.006
QH118B_1 Water Buffaloes: 1-4	.006	.003	.002	.001	.001	.001	.001	.001	.001	.000	.008	.004	.004	.003	.002
QH118B_2 Water Buffaloes: 5-9	.000	0.000	.000	.000	.001	0.000	0.000	0.000	.000	.000	.001	.000	0.000	.000	.001
QH118B_3 Water Buffaloes: 10+	.000	.000	.000	.000	.000	0.000	0.000	0.000	0.000	.000	0.000	.000	.000	.000	.001
QH118C_1 Horses/donkeys/mules: 1-4	.002	.001	.001	.000	.000	.001	.000	0.000	0.000	.000	.003	.002	.001	.001	.001
QH118C_2 Horses/donkeys/mules: 5+	.000	.000	.000	.000	0.000	0.000	.000	.000	0.000	0.000	.000	0.000	.001	0.000	0.000
QH118C_3 Horses/donkeys/mules: 10+	0.000	.000	0.000	0.000	0.000	0.000	0.000	0.000	0.000	0.000	0.000	0.000	.001	0.000	0.000
QH118D_1 Goats/sheep: 1-4	.088	.079	.046	.026	.008	.050	.027	.012	.006	.003	.096	.091	.100	.065	.044
QH118D_2 Goats/sheep: 5-9	.026	.028	.021	.015	.006	.015	.011	.008	.004	.001	.027	.032	.036	.031	.027

QH118D_3 Goats/sheep: 10+	.005	.006	.007	.006	.004	.003	.003	.004	.002	.001	.004	.008	.007	.013	.012
QH118E_1 Pigs: 1-4	.075	.013	.007	.004	.002	.010	.004	.003	.002	.001	.121	.028	.017	.010	.005
QH118E_2 Pigs: 5-9	.007	.002	.001	.001	.001	.001	.000	.000	.001	.000	.010	.005	.002	.003	.002
QH118E_3 Pigs: 10+	.002	.001	.001	.001	.001	.001	.001	.001	.001	.001	.002	.002	.002	.002	.002
QH118F_1 Chickens/poultry: 1-9	.321	.262	.207	.152	.081	.199	.154	.124	.099	.054	.354	.327	.292	.260	.184
QH118F_2 Chickens/poultry: 10-29	.115	.134	.122	.114	.069	.069	.074	.069	.062	.036	.115	.144	.177	.178	.188
QH118F_3 Chickens/poultry: 30+	.007	.013	.017	.022	.027	.007	.009	.014	.019	.010	.005	.012	.016	.024	.053
landarea	.431	.337	.300	.376	.459	.091	.112	.242	.141	.376	.528	.477	.468	.510	.879
